# Periportal steatosis in mice affects distinct parameters of pericentral drug metabolism

**DOI:** 10.1038/s41598-022-26483-6

**Published:** 2022-12-17

**Authors:** Mohamed Albadry, Sebastian Höpfl, Nadia Ehteshamzad, Matthias König, Michael Böttcher, Jasna Neumann, Amelie Lupp, Olaf Dirsch, Nicole Radde, Bruno Christ, Madlen Christ, Lars Ole Schwen, Hendrik Laue, Robert Klopfleisch, Uta Dahmen

**Affiliations:** 1 Experimental Transplantation Surgery, Department of General, Visceral and Vascular Surgery, University Hospital Jena, Jena, Germany; 2 Department of Pathology, Faculty of Veterinary Medicine, Menoufia University, Shebin Elkom, Menoufia, Egypt; 3 Institute for Systems Theory and Automatic Control, Faculty of Engineering Design, Production Engineering and Automotive Engineering, University of Stuttgart, Stuttgart, Germany; 4 Institute for Theoretical Biology, Institute of Biology, Humboldt-University, Berlin, Germany; 5 MVZ Medizinische Labore Dessau Kassel GmbH, Bauhüttenstraße 6, 06847 Dessau-Roßlau, Germany; 6 Institute of Pharmacology and Toxicology, Jena University Hospital, Jena, Germany; 7 Institute of Pathology, Klinikum Chemnitz, Chemnitz, Germany; 8 Cell Transplantation/Molecular Hepatology Lab, Department of Visceral, Transplant, Thoracic and Vascular Surgery, University of Leipzig Medical Center, Leipzig, Germany; 9 Fraunhofer MEVIS, Max-Von-Laue-Str. 2, 28359 Bremen, Germany; 10 Institute of Veterinary Pathology, Freie Universität Berlin, Berlin, Germany

**Keywords:** Metabolic disorders, Experimental models of disease, Hepatology, Gastrointestinal diseases, Liver diseases

## Abstract

Little is known about the impact of morphological disorders in distinct zones on metabolic zonation. It was described recently that periportal fibrosis did affect the expression of CYP proteins, a set of pericentrally located drug-metabolizing enzymes. Here, we investigated whether periportal steatosis might have a similar effect. Periportal steatosis was induced in C57BL6/J mice by feeding a high-fat diet with low methionine/choline content for either two or four weeks. Steatosis severity was quantified using image analysis. Triglycerides and CYP activity were quantified in photometric or fluorometric assay. The distribution of CYP3A4, CYP1A2, CYP2D6, and CYP2E1 was visualized by immunohistochemistry. Pharmacokinetic parameters of test drugs were determined after injecting a drug cocktail (caffeine, codeine, and midazolam). The dietary model resulted in moderate to severe mixed steatosis confined to periportal and midzonal areas. Periportal steatosis did not affect the zonal distribution of CYP expression but the activity of selected CYPs was associated with steatosis severity. Caffeine elimination was accelerated by microvesicular steatosis, whereas midazolam elimination was delayed in macrovesicular steatosis. In summary, periportal steatosis affected parameters of pericentrally located drug metabolism. This observation calls for further investigations of the highly complex interrelationship between steatosis and drug metabolism and underlying signaling mechanisms.

## Introduction

Little is known regarding morphological alteration in distinct lobular zones affecting liver function such as cytochrome p450 (CYP) dependent drug metabolism.

Cytochrome p450 enzymes are a superfamily of proteins catalyzing the oxidation of organic substances, an important step in the detoxification of drugs^1,2^. CYP enzymes occur in all major organs, but predominantly in the liver^2^. Hepatic CYP enzymes are normally expressed in the pericentral region of the hepatic lobule^3^. This is of importance since the various metabolic pathways and functions are subject to spatial organization along the porto-central axis in the liver lobule, a phenomenon called metabolic zonation^4–7^.

It seems obvious that necrosis or damage of the pericentral region will affect pericentral expression as well as the activity of CYP enzymes^8^. However, the impact of periportal damage or alterations on pericentral processes is rather elusive. There is first evidence that periportal alterations can also have an impact on pericentral metabolic processes. Ghallab et al. showed recently that pericentral as well as periportal fibrosis had a similar influence on the zonated expression of hepatic CYP proteins. In both cases, fibrosis caused a mild to complete loss of pericentral CYP expression^8^. However, they did not investigate CYP activity in or ex-vivo.

Diseases such as fatty liver disease may have an impact on the zonated expression of CYP enzymes and possibly also on the zonation of function. For instance, experimental and clinical studies report consistently that CYP3A4 is downregulated in fatty liver in terms of either activity, expression level, or pharmacokinetics (PK)^9–13^. In contrast, most but not all human and animal studies state that CYP2E1 is upregulated in non-alcoholic steatohepatitis in terms of either activity or expression^9,10,14–16^. In contrast, it was also observed in patients that CYP2E1 is downregulated in terms of protein and mRNA expression^17^. Furthermore, in rats subjected to a methionine choline-deficient (MCD) diet, CYP2E1 was downregulated in terms of activity and mRNA expression^12^.

Taken together, the impact of steatosis on drug metabolism is discussed controversially^18^ (see Supplementary Table S2 for details). So far not much attention was paid to a clear description of severity, type, and zonation of fat accumulation. These factors are possibly affecting CYP expression pattern and/or CYP activity and consequently CYP mediated function such as drug metabolization.

The objective of our work was to study the impact of periportal steatosis on pericentral drug metabolism focusing on the important CYP isoforms CYP1A2, CYP2D6, CYP2E1, and CYP3A4. Our study is unique in respect to relating zonal distribution and pattern of steatosis to key enzymes of drug metabolism. As stated above, most other drug metabolism studies do not take this potential impact of steatosis into account (Supplementary Materials, Table S3). Furthermore, either zonal distribution of CYP enzymes is investigated or overall activity and/or expression levels. In other words, studies focusing on zonal distribution of CYP-expression pattern did not involve hepatic metabolic disorders like fatty liver and their impact on CYP-mediated drug metabolism.

Hence, studying CYP zonation in the complex relationship between steatosis and drug metabolism is a new and unknown aspect. However, this aspect seems to be of importance, since there is accumulating evidence that periportal morphological changes such as fibrosis may affect certain features of the pericentrally located drug metabolism^8^. Our study was thus designed to investigate this relationship.

Therefore, mixed micro- and macrovesicular periportal steatosis of various degrees were induced using a high-fat diet with reduced methionine choline content (HF-diet). To quantify the relationship between steatosis and CYP catalyzed drug metabolism: In the first step, hepatic steatosis was characterized and quantified. In the second step, we analyzed the spatial distribution of drug-metabolizing enzymes. In the third step, the CYP activity ex-vivo was assessed and the correlation between activity and steatosis severity and steatosis pattern were determined. Finally, a pharmacokinetic study to assess the CYP activity in vivo was performed based on a drug cocktail of midazolam for CYP3A4, caffeine for CYP1A2, and codeine for CYP2D6^19^. Pharmacokinetic parameters were calculated from the drug elimination curves and the association between severity and pattern of steatosis were analyzed.

## Results

### Characterization and quantification of hepatic steatosis

#### Dietary induction resulted in mixed steatosis predominantly confined to the periportal and midzonal area

The feeding protocol induced predominantly periportal to midzonal steatosis, but no weight loss. Interestingly, we observed a rather heterogeneous distribution within and in between liver lobes (for details, see Supplementary Fig. S1).

In animals fed for 2 weeks, we observed a mixed, but predominantly microvesicular steatosis pattern. In the immediate surrounding of the portal tract, there was a small rim of hepatocytes with large fat vacuoles. However, the majority of hepatocytes in the outer periportal zone and the midzonal area contained microvesicular lipid droplets. After four weeks of feeding, the ratio of microvesicular to macrovesicular steatosis pattern was reversed. The hepatocytes in the periportal zone and part of the midzonal area were filled with large fat vacuoles. In contrast, there was a rather small rim of hepatocytes in the midzonal to the pericentral area which contained small lipid vesicles.

#### Biochemical assessment of severity showed a similar load of triglycerides (TG)

**Figure 1:**
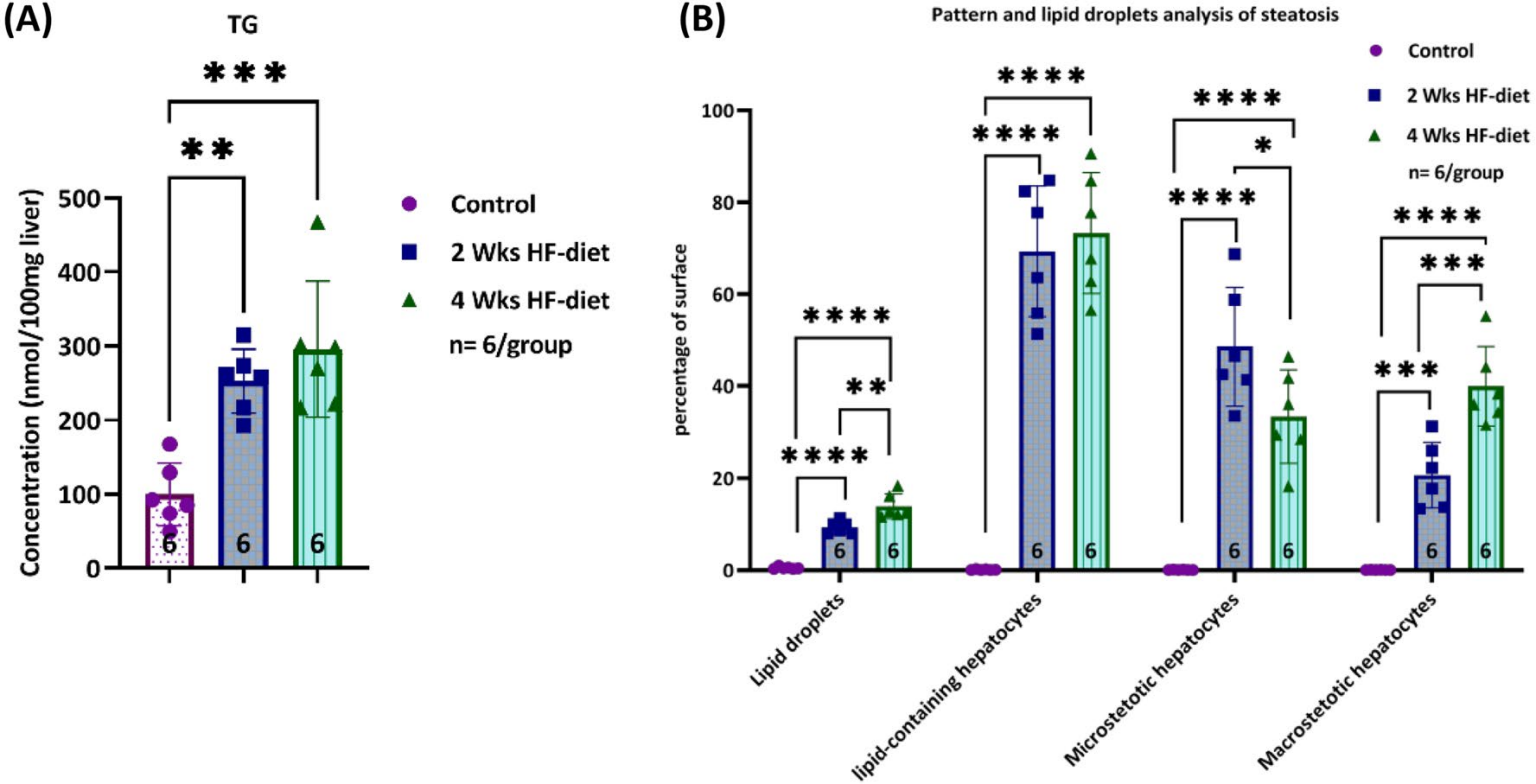
Severity of hepatic steatosis. (**A**) Total triglyceride depending on diet. Total triglyceride measurement showed significantly higher levels in the HF-diet groups compared to control, but no differences between the groups subjected to two respectively four weeks of HF-diet; (**B**) Lipid droplets, lipid-containing hepatocytes, microsteatotic hepatocytes, and macrosteatotic hepatocytes depending on diet. Image analysis using different algorithms detected specific differences in severity when subjecting animals either to 2 or 4 weeks of feeding the special diet. Lipid droplet analysis revealed a significantly higher surface covered by the droplets. Pattern analysis assessing micro-and macrovesicular steatosis separately revealed a strikingly and significantly larger surface covered by microvesicular steatotic hepatocytes after two weeks of feeding in contrast to the significantly larger surface covered by macrovesicular steatotic hepatocytes after four weeks of feeding. The total surface covered by steatotic hepatocytes was similar in both groups (*significance level < 0.05, **significance level < 0.01, ***significance level < 0.001, ****significance level < 0.0001, sample size of each group displayed in the bottom of the bar).

Application of the steatosis-inducing diet caused a substantial and significant increase in the mean TG concentration per 100 mg homogenized liver. In the experimental groups, the TG concentration was at least twice as high as in the control group. We observed 192.7 nmol/100 mg tissue as the lowest value in the two experimental groups compared to a mean of 99.6 ± 42.3 nmol/100 mg tissue in the control group, resulting in a highly significant difference between control and experimental groups (p = 0.0002), see Fig. 1A.

The mean TG concentration in the livers of animals subjected to four weeks of feeding was higher compared to the animals subjected to two weeks of feeding (with 296.1 ± 91.6 nmol/100 mg of tissue after four weeks compared to 252.8 ± 43.0 nmol/100 mg tissue after two weeks. However, the difference between the two experimental groups did not reach statistical significance (p = 0.48).

#### Histological assessment revealed a transition from predominantly micro- to predominantly macrovesicular steatosis over time

For the histological quantification of steatosis severity and pattern, we applied two different image algorithms.

First, we analyzed the relative surface covered by lipid droplets as done frequently in image analysis-based assessment of severity^20,21^. In animals subjected to two weeks of feeding, the relative surface covered by the lipid droplets ranged between 8 and 11%. In contrast, in animals fed for four weeks, the relative surface covered by lipid droplets was significantly larger (p = 0.001), albeit the absolute difference in “relative surface” was not so high (9.3 ± 1.3% versus 13.9 ± 2.7%)%, see Fig. 1B. However, this type of analysis is underestimating the impact of microvesicular steatosis, since even a high number of hepatocytes with small fat vacuoles is not contributing to the relative surface substantially.

Second, we determined the relative surface covered by lipid-containing hepatocytes, an analysis closer to the conventional histopathological assessment applied clinically. Clinicians estimate the relative number of hepatocytes containing fat droplets, irrespectively of the size^22^ with < 33% considered to represent mild steatosis, 33–66% reflecting moderate steatosis and > 66% indicating severe steatosis. In contrast to the rat model^23,24^, The 2 weeks feeding period already induced severe steatosis with the relative surface covered by fat-laden hepatocytes exceeding 66%, see Fig. 1B. Using the pattern recognition algorithm for calculating the total area covered by fat-laden hepatocytes, the difference between the two groups subjected to either 2 weeks or 4 weeks of induction was not significant, as already observed in the TG analysis.

However, using this pattern recognition algorithm, we were able to discriminate between micro-and macrovesicular steatosis. As described above, the shorter feeding period was associated with a predominantly microvesicular steatotic pattern. About 48.6 ± 12.9% of the relative surface was covered with microvesicular steatotic hepatocytes whereas 20.7 ± 7.1% was taken by macrovesicular steatotic hepatocytes, representing a ratio of micro- to macrovesicular steatosis of 2.4:1. In contrast, the longer induction period resulted in a predominantly macrovesicular steatotic pattern. After 4 weeks of feeding, a significantly smaller proportion of the relative surface (33.4 ± 10.2%) was covered with microvesicular steatotic hepatocytes, whereas a significantly larger proportion was taken by macrovesicular steatotic hepatocytes (39.9 ± 8.6%), resulting in a ratio of 0.8:1. In other words, the ratio of micro- to macrovesicular steatosis changed substantially.

This pattern reflects the development of steatosis starting with the accumulation of tiny lipid droplets in the periportal area and extending towards the pericentral zone. Over time and accumulation of more fat in the hepatocytes, the droplets became larger, resulting in macrovesicular steatosis, as reported previously^25^.

### CYP expression

#### Steatosis did not affect zonal distribution and extent of CYP expression

As expected, all CYP enzymes were expressed in the pericentral region, albeit to a different extent.

**Figure 2:**
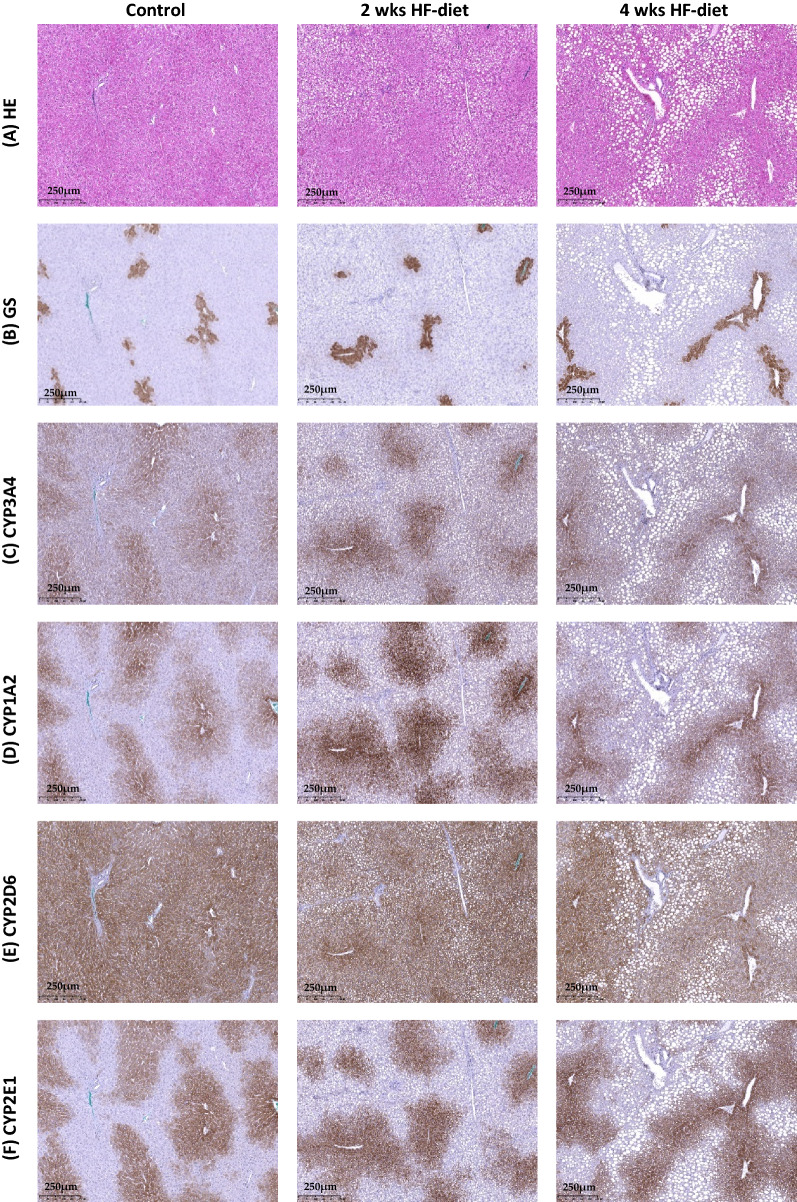
Visualization of steatosis and CYP expression in normal and experimental animals subjected to two weeks respectively four weeks of HF-diet. (**A**) HE-staining shows periportal steatosis with predominantly microvesicular pattern after two weeks of feeding and predominantly macrovesicular pattern after four weeks of feeding; (**B**) Glutamine synthetase (GS)-staining for annotation of pericentral hepatocytes around the central vein, and discrimination of periportal from pericentral zones; (**C**) CYP3A4 staining present in pericentral location without substantial differences in the distribution of positive signals between the three groups; (**D**) CYP2D6 staining also present in pericentral location without substantial differences in the distribution of positive signals between the three groups; (**E**) CYP2D6 expressed almost in the whole lobules with 1–2 lines of dark brown stained hepatocytes around the central vein with similar pattern in all three groups; (**F**) CYP2E1 expression slightly different compared to CYP3A4 with 4–5 lines of hepatocytes presenting signals of strong intensity around the central vein, also with no differences between groups; Scale bar 250 µm.

**Figure 3:**
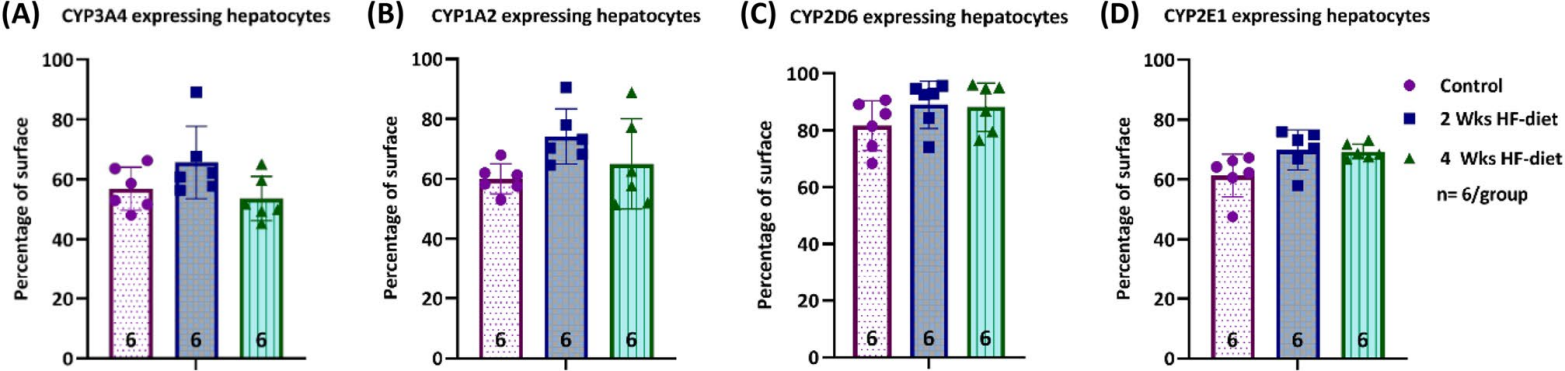
Quantification of CYP-expression using the pattern recognition algorithm. (**A**–**D**) No significant difference in CYP3A4, CYP1A2, CYP2D6, and CYP2E1 expression pattern in terms of relative surface covered by positively stained hepatocytes, sample size of each group displayed in the bottom of the bar.

CYP3A4 staining was visible in the first 2–3 lines of hepatocytes surrounding the central vein and moderate signal intensity in the pericentral area of the lobule. When calculating the relative surface covered by strong and moderate signals, we did not find a difference in CYP3A4 surface between the three groups, see Figs. 2 and 3.

CYP1A2 expression followed a similar pattern as CYP3A4 with a strong signal in the first 2–3 lines of perivenous hepatocytes and a moderate signal extending throughout the pericentral third of the lobule. Here also the pattern and extension were not affected by the steatosis-inducing diet, see Figs. 2 and 3.

CYP2D6 was expressed almost in the complete lobules. However, signal intensity was strongest around the central vein. We observed 1–2 lines of dark brown stained hepatocytes around the central vein. The remaining hepatocytes showed a moderate signal throughout the lobules. The CYP2D6 pattern as well as the extension were also not affected by the steatosis-inducing diet, see Fig. 2 and 3.

The pattern of CYP2E1-staining was slightly different compared to CYP3A4. In both control and experimental groups, we observed 4–5 lines of hepatocytes presenting signals of strong intensity around the central vein. Also, for CYP2E1, the pattern and extension was not affected by the steatosis-inducing diet, see Figs. 2 and 3.

### Ex-vivo activity of selected CYP-enzymes

**Figure 4:**
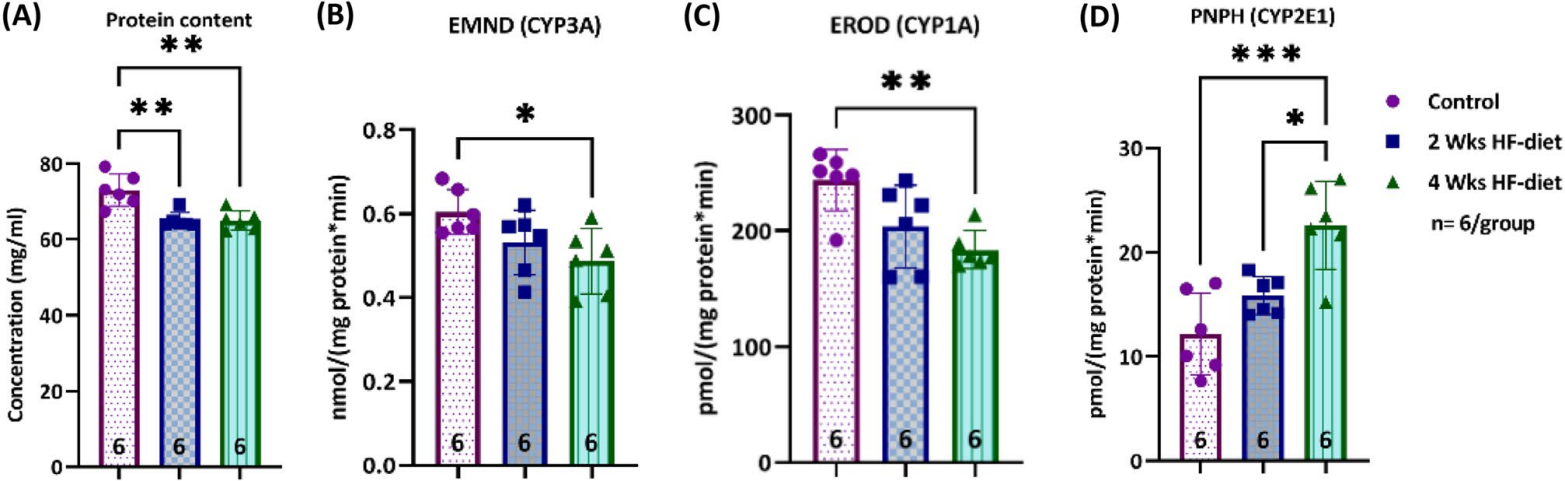
CYP activity. (**A**) Protein concentration in liver samples was statistically significantly lower in steatotic samples compared to control, but similar in the two experimental groups; (**B**) CYP3A activity determined with the EMND assay was significantly lower in the samples from animals with severe steatosis as determined by the lipid droplet analysis; (**C**) The results of the EROD assay for CYP1A activity showed significantly lower activity in the samples from animals with severe steatosis; (**D**) The PNPH assay for CYP1E1 activity showed increasing activity with increasing steatosis severity (*significance level < 0.05, **significance level < 0.01, ***significance level < 0.001, ****significance level < 0.0001, sample size of each group displayed in the bottom of the bar).

The CYP activity in liver tissue was measured using four model reactions covering different combinations of enzymes. We first determined the protein content per gram of liver and then related the activity to mg protein in the tissue. We observed that the relative protein content of the normal control samples ranged between 67.4 and 79.2 mg/ml, see Fig. 4A. In contrast, the relative protein content of the steatotic samples was significantly lower and ranged between 64.0 to 69.2 mg/ml after 2 weeks of feeding and 62.2 to 69.2 mg/ml after 4 weeks of feeding.

Feeding duration had an impact on the activity of CYP3A (EMND assay), CYP1A (EROD assay), and CYP2E1 (PNPH assay), see Fig. 4, but not on ECOD and PROD, see Fig. S2. In the first step of the analysis, we explored the impact of feeding duration on the CYP activity. CYP3A activity measured by the EMND reaction as well as CYP1A activity determined by the EROD reaction were significantly lower in livers from animals subjected to 4 weeks of feeding compared to control tissue from normal animals (p = 0.029, respectively p = 0.005), see Fig. 4B,C. In contrast, the activity of CYP2E1 was almost twice as high in the livers obtained from animals after long term feeding compared to control (p = 0.0003), see Fig. 4D.

**Figure 5:**
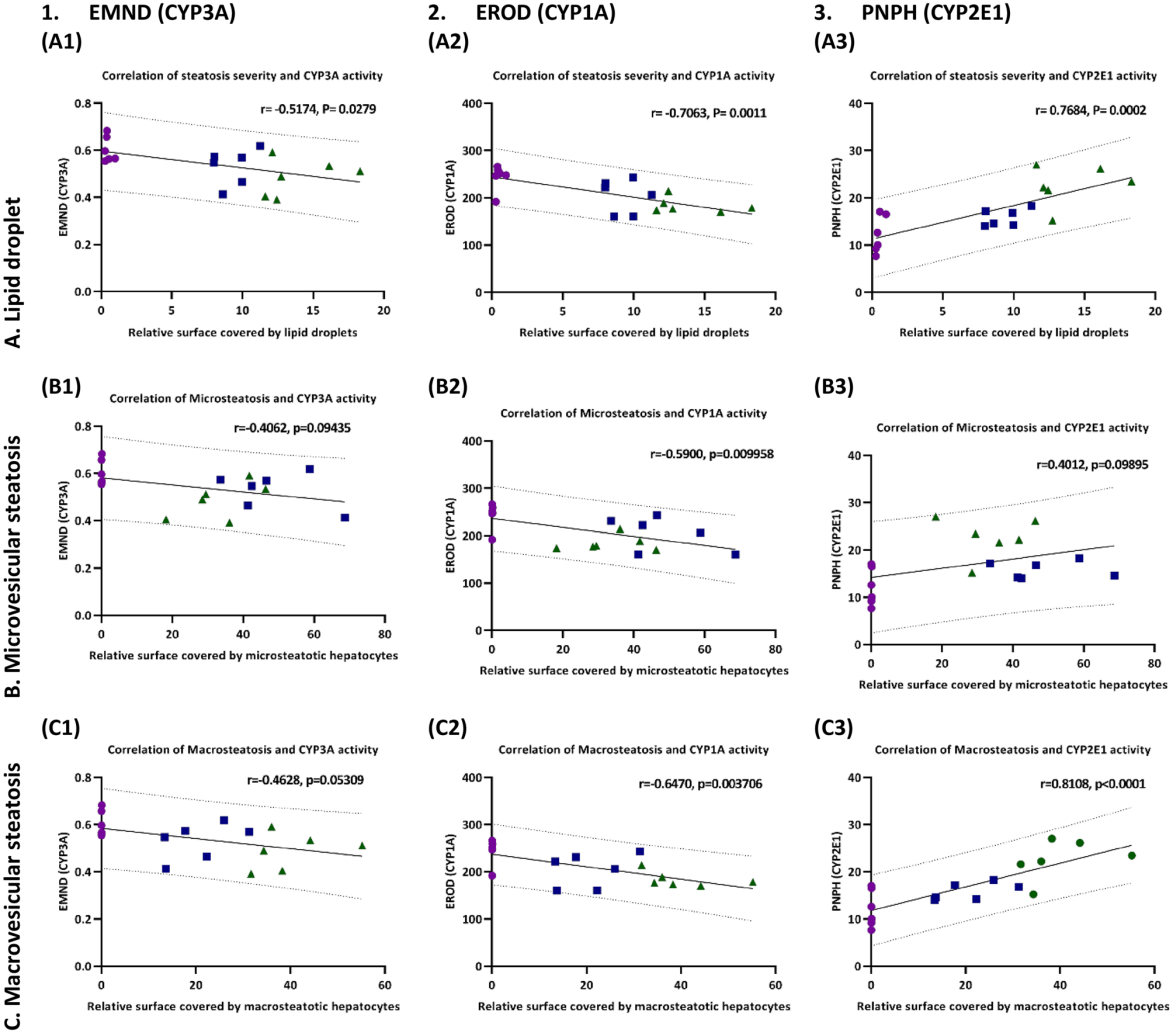
Correlation between steatosis severity and CYP activity. (Row A) Severity of steatosis as determined by lipid droplet analysis in percent of surface; (Row B) Extent of microvesicular steatosis, in percent of surface; (Row C) Extent of macrovesicular steatosis, expressed in percent. (Column 1) Correlation of steatosis type and severity with activity of CYP3A; (Column 2) Correlation of steatosis type and severity with activity of CYP1A; (Column 3) Correlation of steatosis type and severity with activity of CYP2E1. Control as magenta circles, two weeks HF-diet as blue squares, 4 weeks HF-diet as green triangles.

The severity of steatosis affected ex-vivo activity of CYP1A2 and CYP2E1. In the second step of the analysis, we used the lipid droplet results of the individual animals to calculate the linear correlation between the severity of steatosis and the CYP activity. As expected from the first analysis, we found a moderate negative correlation between the relative surface covered by lipid droplets and CYP3A activity as measured in the EMND assay, see Fig. 5A1. We observed a strong negative correlation with CYP1A activity (EROD assay), see Fig. 5A2. We also confirmed the strong positive correlation between steatosis severity and CYP2E1 activity as determined in the PNPH assay, see Fig. 5A3.

The pattern of steatosis influenced the ex-vivo activity of CYP1A and CYP2E1. In the third step, we analyzed the correlation between the predominant pattern of steatosis and the CYP activity. In contrast to the droplet analysis, the extent of microvesicular steatosis (%) was not significantly correlated to the results of EMND and PNPH reflecting mostly the activity of CYP3A and CYP2E1, see Fig. 5B1,B3, but showed a moderate negative correlation to the CYP1A activity, see Fig. 5B2. Furthermore, the extent of macrovesicular steatosis was not significantly correlated to CYP3A, moderately correlated to CYP1A, but showed a strong positive correlation to CYP2E1 activity.

Taken together, certain features of periportal steatosis had a strong impact on the activity of CYP enzymes located in the pericentral zone of the liver lobule. The severity of steatosis was negatively correlated to the activity of CYP3A and CYP1A, but positively to the activity of CYP2E1. Besides the severity, the pattern of steatosis also had an impact on CYP activity. Macrovesicular steatosis was negatively correlated to the activity of CYP1A but positively correlated to the activity of CYP2E1. These observations suggest a highly complex interplay between an alteration in the periportal zone and the molecular response in the pericentral region.

### Pharmacokinetic study (to assess the in-vivo activity of CYP enzymes)

First, we looked at the drug elimination curves in all three experimental groups. Second, we determined the pharmacokinetic parameters such as peak time, peak concentration, and half-life of all drugs (see Supplementary Table S4) and compared the AUC of the control and experimental groups. Third and fourth, we investigated the impact of steatosis severity and steatosis pattern on the AUC by calculating the linear correlation.

**Figure 6:**
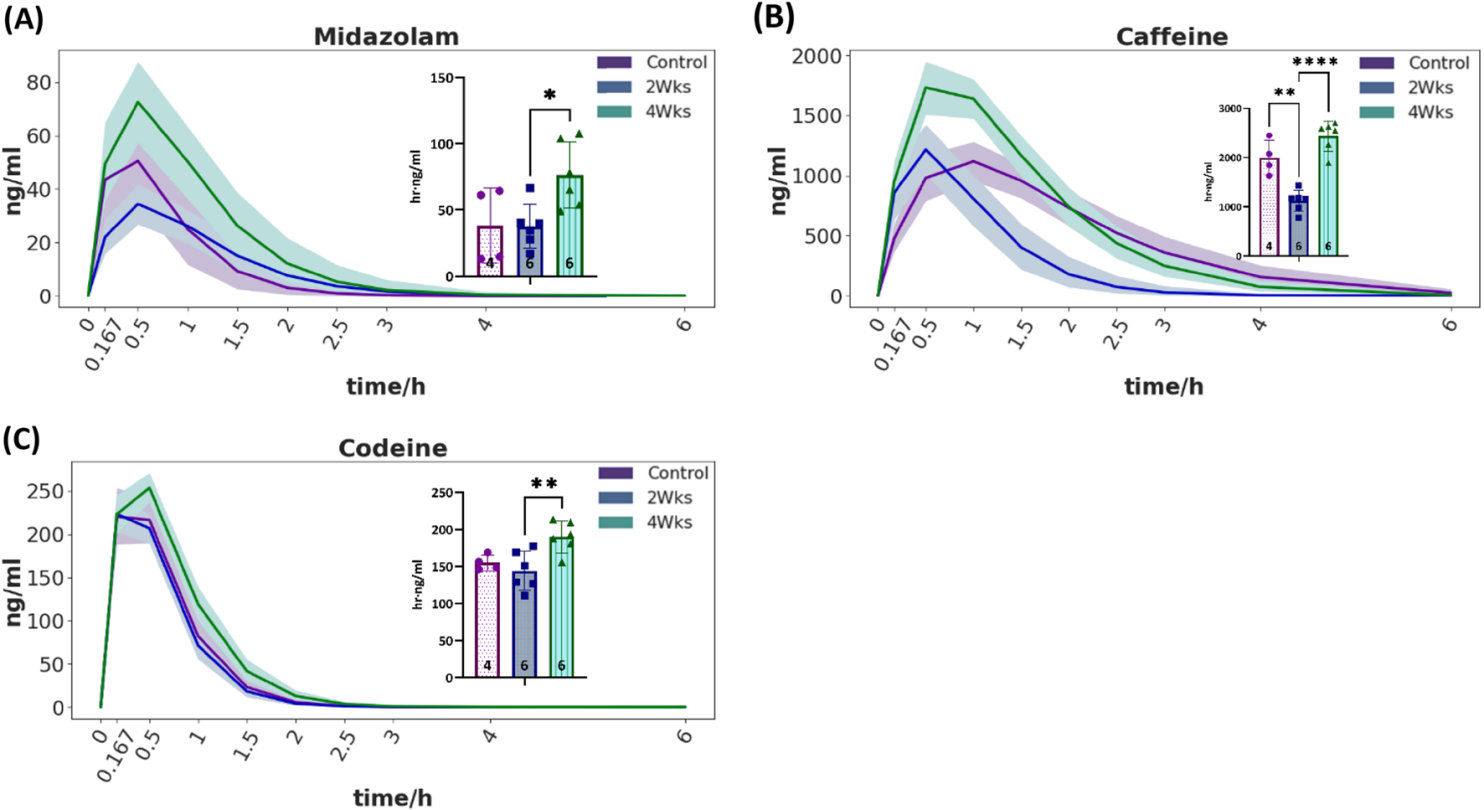
Drug elimination curves of the test drugs and metabolites and corresponding AUC. (**A**) Midazolam; (**B**) Caffeine; (**C**) Codeine; *significance level < 0.05, **significance level < 0.01, ***significance level < 0.001, ****significance level < 0.0001, sample size of each group displayed in the bottom of the bar. Solid lines are mean, shaded areas correspond to the 95% credibility interval from the Bayesian analysis.

The pharmacokinetic analysis revealed a similar exponential elimination curve for all three compounds, as expected. Peak concentration appeared within 15–60 min and the elimination of the drug occurred within 4–6 h, see Fig. 6, for metabolites see Supplementary Fig. S4. The shaded area illustrates the *95%* credibility interval from the Bayesian analysis. Non-overlapping credibility intervals indicate a significant difference in the dynamics at this given time point. However, it is difficult to predict an overall different behavior when credibility intervals overlap partly in time.

Feeding duration had an impact on the AUC of all three test drugs. We calculated the pharmacokinetic parameters and checked for statistically significant differences between the experimental groups. To make conclusive statements about significant overall differences between conditions, we investigated AUC values as an aggregate measure for the time courses. This parameter encompasses the time to peak concentration, the peak concentration (Cmax), and the half-life, and therefore seemed most suitable for in-depth analysis.

Two weeks of feeding, resulting in predominantly microvesicular steatosis, accelerated the elimination of caffeine as indicated by the smaller AUC. Interestingly, the animals seemed to become somehow “tolerant” to this effect since the AUC reached normal levels in the animals subjected to four weeks of this diet. In contrast, four weeks of feeding, resulting in predominantly macrovesicular steatosis, decelerated the elimination of midazolam and codeine as indicated by the larger AUC.

**Figure 7:**
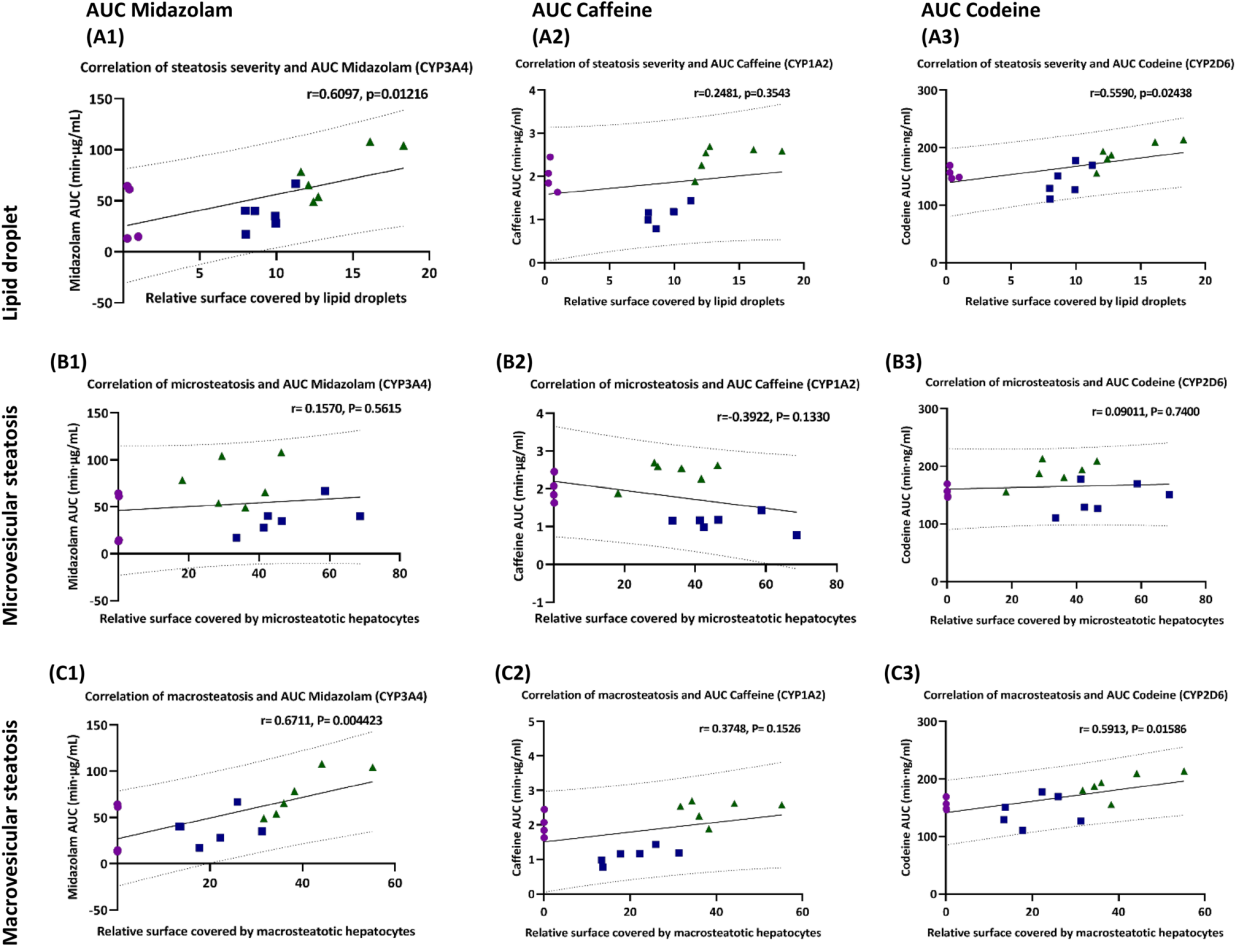
Correlation between steatosis severity and AUC (A-C). (Row A) Severity of steatosis as determined by lipid droplet analysis in percent of surface; (Row B) Extent of microvesicular steatosis, in percent of surface; (Row C) Extent of macrovesicular steatosis, expressed in percent. (Column 1) Correlation of steatosis type and severity with AUC of Midazolam; (Column 2) Correlation of steatosis type and severity with AUC of caffeine; (Column 3) Correlation of steatosis type and severity with AUC of codeine. Control as magenta circles, two weeks HF-diet as blue squares, four weeks HF-diet as green triangles.

The severity of steatosis correlated with the AUC of midazolam and codeine. Next, we analyzed the correlations between the severity of steatosis of individual animals irrespective of the feeding time with the corresponding AUC, see Fig. 7 for the parent drugs and Figs. S5, S6, and S7 in the supplement for the metabolites. The severity of steatosis showed a moderate positive correlation with the AUC of midazolam and codeine, suggesting the decelerated elimination of both test drugs, see also Fig. 7, row A and row C. As expected, based on the results of the group analysis, we did not find a correlation between the severity of the transiently appearing microvesicular steatosis and the AUC of all three test drugs, see Fig. 7, row B.

The pattern of steatosis also correlated with the AUC of midazolam and codeine. Similar results were obtained when correlating the steatosis patterns with the AUC. The macrovesicular steatosis pattern showed a moderate positive correlation with the AUC of midazolam and codeine, suggesting the decelerated elimination of midazolam and codeine, see Fig. 7, row A and row C.

### Summary of correlation analysis

**Figure 8:**
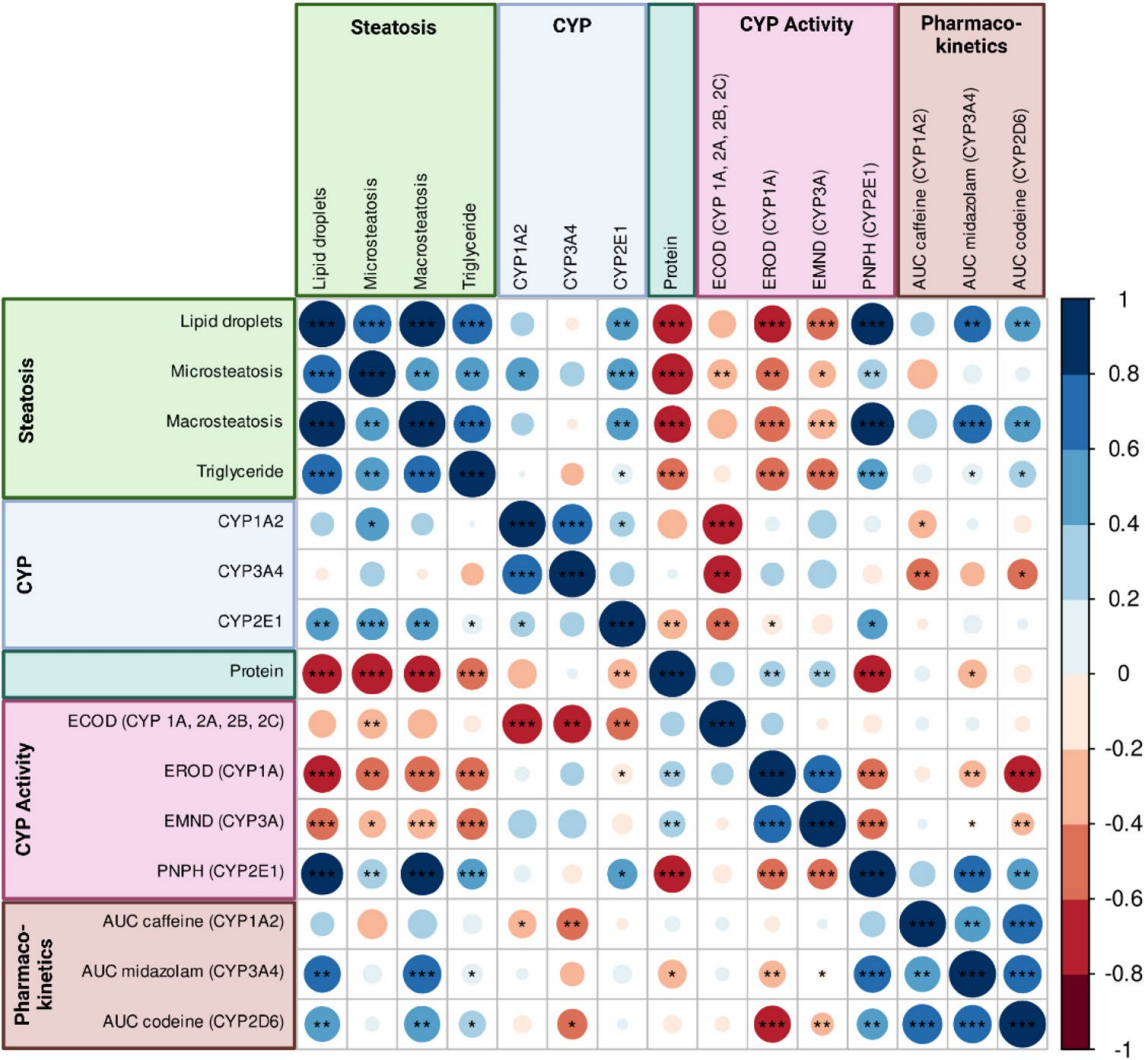
Correlation analysis. Correlation matrix based on Pearson correlation with positive correlation in blue and negative correlation in red. Areas of the circle are proportional to the correlation coefficient. Significance levels are *0.05, **0.01, and ***0.001, with p-values adjusted for multiple testing using Benjamini and Hochberg. Features have been sorted by category.

To provide a concise overview of the main results and observed associations we performed a systematic correlation analysis, see Fig. 8.

In summary, (a) variables corresponding to steatosis all showed a highly significant positive correlation to each other (lipid droplets, microvesicular steatosis, macrovesicular steatosis, triglyceride content). (b) no significant correlations could be observed between steatosis variables and the distribution of CYP expression as indicated by the CYP3A4 and CYP1A2 covered surface, whereas a small positive correlation existed in the case of CYP2E1. (c) Protein content showed a strong negative association with steatosis parameters. (d) CYP activity measured via EROD (CYP1A) and EMND (CYP3A) showed a negative association with steatosis parameters whereas PNHP (CYP2E1) showed a strong positive association. (e) Lipid droplets and macrosteatosis were positively correlated with the AUC of midazolam and codeine.

## Discussion

Our study resulted in four novel findings. (1) Dietary induction using a high-fat diet with low methionine content resulted in mixed periportal steatosis, heterogeneously distributed throughout the liver. (2) Severity and pattern of periportal steatosis were not associated with changes in the zonal distribution and the extent of CYP expression. (3) Severity and pattern of periportal steatosis were associated with changes in the ex-vivo activity of CYP1A, CYP3A, and CYP2E1. (4) Severity and pattern of periportal steatosis were associated with changes in the pharmacokinetics of caffeine (CYP1A2) and midazolam (CYP3A4), but not of codeine (CYP2D6).

We were interested in studying the impact of a clearly defined zonated distribution of steatosis on pericentrally located processes. Therefore, we selected a dietary model of periportal steatosis to investigate the effect on drug metabolism, one of the key processes taking place in the pericentral zone.

We used a high-fat diet with reduced methionine and choline to induce periportal hepatic steatosis. Using this diet in rats, periportal steatosis developed within one week of feeding^26^. Extension to the midzonal region was seen within two weeks of feeding. Severe steatosis spanning from the periportal to the pericentral region was induced within four weeks of feeding. In contrast, mice did react slower to this induction protocol, as expected based on a recent review of Zhong^27^. He concluded that rats were more susceptible to a high-fat diet than mice, as indicated by a faster progression of the histological alterations. of the histological alterations.

Furthermore, mice showed a slightly different pattern of steatosis compared to rats. Mice were not only showing macrovesicular steatosis but also microvesicular steatosis. This microvesicular pattern was more pronounced after two weeks compared to four weeks of feeding. This finding is in line with the concept that steatosis seems to develop from the accumulation of small lipid droplets (microvesicular steatosis) in a few hepatocytes^25,28^. At some point, the micro-vesicles fuse to form one large vacuole giving the fat-laden hepatocyte the typical signet-ring appearance. Therefore, macrovesicular steatosis is considered to be the result of a rather chronic process.

However, other reasons exist for microvesicular steatosis. Microvesicular steatosis can occur as a result of an acute or toxic insult to the liver^29^. It is also observed in the context of drug-induced liver injury^30^. According to Silva, microvesicular steatosis may develop due to an impairment of the mitochondrial beta-oxidation of fatty acids, suggesting a mitochondrial disorder^31^ potentially affecting metabolic function.

Grading the severity of hepatic steatosis followed four complementary approaches. The biochemical quantification of TG and quantification of the relative surface of fat-laden hepatocytes discriminated clearly between normal and steatotic livers. Discrimination between the two steatosis induction protocols was enabled by the lipid droplet analysis. Lipid droplets analysis is used frequently^24,32–34^ and is based on the identification of white area of the lipid droplets compared to the remaining area. However, accuracy is hampered in the case of microvesicular steatosis, since the small droplets cannot be identified unambiguously. Therefore we decided to train the pattern recognition algorithm for quantification of micro- respectively macrosteatosis separately, an approach comparable^22,35^ but more precise than clinical routine diagnosis. Pathological assessment of the severity is based on the estimation of the relative percentage of hepatocytes with a lipid droplet^22,36^. However, pathologists must also report zonal distribution (periportal versus pericentral steatosis) and the steatosis pattern (macrovesicular versus microvesicular steatosis), but only qualitatively.

Our study revealed three new observations elucidating the impact of periportal steatosis on drug metabolism, which deserve further attention. As outlined above, periportal steatosis did not affect the pericentral expression pattern of the four key CYP enzymes, which were studied. However, periportal steatosis did affect the activity of CYP1A, CYP2E1, and CYP3A. Periportal steatosis did also affect the pharmacokinetics of caffeine (CYP1A2) and midazolam (CYP3A4), but not of codeine (CYP2D6).

The impact of steatosis pattern or zonal distribution on CYP expression level and distribution is not well-investigated. None of the published drug metabolism studies disclosed these features (Supplementary Materials, Tables S1 and S2) when describing their results. Interestingly we did not observe any effect of steatosis, irrespectively of severity or pattern, on the distribution of CYP-protein expression. Other studies reported a decreased expression level in steatotic livers from human patients^17^, rats^12,37^, and mice^38^ as demonstrated by western blot or qPCR (Supplementary Materials, Table S3). However, they did not investigate the extent of zonal distribution using immunohistochemistry. It is possible that zonation remains unaffected, but total mRNA and protein expression levels may be reduced in a severely steatotic liver. To clarify this issue, complementary techniques are needed. The spatial distribution must be visualized and quantified using immunohistochemistry, and protein respectively mRNA expression must be quantified using western blot or qPCR.

Nevertheless, changes in mRNA levels do not necessarily reflect changes in protein levels and may not be predictive of protein activity due to posttranscriptional, translational and posttranslational regulatory processes^39^.

Looking at the mechanism underlying the regulatory processes of CYP expression, taking distribution and activity into account adds further complexity. As an example, upregulation of CYP2E1 in fatty liver disease is attributed to different mechanism as summarized in a recent review by Massart et al.^40^. In alcoholic liver disease, the higher CYP2E1 levels are thought to be the result of the inhibition of degradation by ethanol. The mechanism in non-alcoholic fatty liver disease is less clear. A vicious cycle was postulated, starting with increased levels of fatty acids and insulin resistance promoting the expression of CYP2E1. The increased levels of CYP2E1 in return may induce lipid peroxidation, oxidative damage and aggravate insulin resistance, which is augmenting the fat accumulation in the liver.

However, the studies reporting an impact of steatosis on CYP expression levels used other animal models of steatosis, a factor also affecting comparability of the results. Stärkel et al. observed in MCD-fed rats a decrease in CYP2E1 expression and activity^12^. In contrast, Zhang et al. used the HF-diet model and observed a reduced activity and mRNA expression of phase I enzymes CYP1A2, CYP2B1, CYP2C11, CYP3A1, and CYP4A1. Interestingly, he did not observe any impact on CYP2E1 expression in the liver of obese rats^37^ (Supplementary Material, Table S3).

In conclusion, regulation of CYP expression levels, distribution and activity in steatosis seems to be rather complex as it might be related to the etiology respectively the animal model used.

Nevertheless, there are certain liver pathologies, where zonation and staining intensity of the CYP protein is affected by the underlying liver disease**.** This is obviously the case when the vitality of pericentral hepatocytes is affected as commonly occurs after intoxication. CCl4-induced toxic liver injury with pericentral necrosis caused a decrease in the expression and staining intensity of CYP proteins^19^. However, alteration of CYP expression may also occur in the case of periportal morphological impairment. The best example is the study of Ghallab who observed a loss of CYP expression not only in the case of pericentral but also in the case of periportal fibrosis^8^. But, morphological damage is not necessarily a prerequisite for an altered CYP expression. Despite normal morphology, selective loss of CYP1A mRNA and protein expression was observed in a mouse model of chronic intermittent hypoxia mimicking sleep apnea^41^.

These observations suggest cellular or molecular events in the periportal region might result in a molecular response of cells in the pericentral regions. As outlined before, Ghallab postulated that in the case of periportal fibrosis inflammatory mediators might provoke the cellular response of pericentrally located hepatocytes, similar as observed in the case of pericentral fibrosis^8^.

Periportal steatosis did have an impact on the activity of selected CYP enzymes, although the expression pattern remained unchanged. Here we observed that the activity of the CYP1A and CYP3A family in liver tissue was downregulated with increasing severity whereas the activity of CYP2E1 was upregulated. These observations are in line with the findings of other groups as summarized in a recent review of Cobbina^18^ and are further confirmed by our literature work-up presented (Supplementary Material, Table S3). However, these studies focused on the impact of steatosis itself but did not investigate different severities, pattern, or zonated distribution.

The activity of enzymes from the CYP3A family was downregulated in the case of steatosis, even across species: in humans with NASH^17,42,43^ as well as in mice subjected to HFD^42^, and rats^44^. Similarly, others also observed that the activity of enzymes from the CYP1A family was downregulated, again in humans^17^, and rats^37^, but not yet in mice.

Few studies were investigating the steatosis-related modulation of CYP2E1 activity. Most studies focusing on CYP2E1 investigated the mRNA or protein expression. Interestingly, CYP2E1 activity seems to be related to the dietary regimen. Stärkel compared two different dietary models and observed an increased activity when applying 5% orotic acid, but a reduced activity when feeding the animals with an MCD diet for 2–6 weeks^12^. In the latter case, the concomitant pronounced inflammation seemed to have a negative impact on the activity.

In our analysis, we correlated the extent of micro- respectively macrovesicular steatosis with the CYP activity and observed striking differences between the two patterns with respect to the activity of CYP2E1. Microvesicular steatosis did not affect CYP2E1 activity. In contrast, predominantly macrovesicular steatosis was correlated positively to the CYP2E1-activity. This finding is in contrast to the one of Stärkel who described a down-regulation of CYP2E1 after inducing macrovesicular steatosis in rats subjected to the MCD diet^12^. Based on these observations, the role of etiology, pattern of steatosis, or concomitant inflammation needs to be further defined.

However, these considerations are limited by the difficulty to distinguish and evaluate enzyme activities corresponding to the expression of each isoform. Nevertheless, the zonal distribution in the expression of each isoform in mice should be closely followed to clarify the relationship with the steatosis severity. Little is known about eventual cross reactivity of the isoform antibodies used.

In contrast, one of the five activity assays is predominantly measuring the activity of one isoform, as shown in the modified Table 3. The PNPH assay is predominantly assessing the activity of one unique Isoform, the CYP 2E1. The EROD assay is assessing the activity of the subfamily 1A with its major isoform 1A2. The PROD assay is used for assessing the activity of the CYP2B family with the key isoforms 2B6 and 2B1. In contrast, the EMND assay is broader and covers the subfamily CYP3A with its major isoforms 3A4, 3A1 and 3A2 and further isoenzymes such as Cyp3a11, 3a13, and 3a16^45^. The ECOD assay is also rather broad by measuring the combined activity of the isoforms 1A1, 1A2, 2A1, 2A6, 2B1, 2B6, 2C9, and 2C11.

Furthermore, the typically used test drug midazolam is also metabolized by other CYP isoenzymes belonging to other subfamilies^46^.

Therefore, it is indeed difficult to clearly attribute the enzyme activities to the expression of each isoform and relate it to the severity of steatosis.

However, we can clarify for CYP2E1 that the expression pattern was not affected by steatosis, whereas the activity was positively correlated to the severity of steatosis reaching high significance (p < 0.001), when comparing severely steatotic with control samples. Likewise, we can state that the expression pattern of CYP1A2 was similar in all three groups, whereas the activity was negatively correlated to the severity of steatosis reaching moderate significance when comparing severely steatotic with control samples. However, we did not investigate the zonal protein expression levels of each isoform in mouse liver, as done in a technically very challenging study of Tachikawa et al.^47^.

We also investigated the impact of severity and pattern of steatosis on the pharmacokinetics of three test drugs. We observed different effects of each drug. Comparison of our results to other studies is difficult since most of them are not characterizing steatosis in great detail. Nevertheless, in previous studies^38,42,48–50^, the impact of steatosis on drug metabolism has been discussed controversially. This suggests that a multitude of factors besides the presence or absence of steatosis may influence drug metabolism.

Predominantly microvesicular steatosis seemed to accelerate caffeine pharmacokinetics as indicated by the smaller AUC. However, this effect was transient. Once the animals developed predominantly macrovesicular steatosis, the elimination was decelerated back to normal as indicated by the statistically significantly larger AUC. In contrast, using a model with an even more severe macrovesicular steatosis in ob/ob mice fed with an MCD diet, Li reported a significantly higher AUC for caffeine compared to the control^38^. Based on these observations, caffeine metabolism reflecting the activity of CYP1A2 seems to be affected strongly by the pattern of steatosis with opposing effects.

In contrast, the PK of midazolam and codeine was not affected by microvesicular steatosis. However, in the case of predominantly macrovesicular steatosis, elimination of midazolam was decelerated, as indicated by the significantly larger AUC compared to the group with microvesicular steatosis. Using a different steatosis induction protocol, Li and coworkers did not observe an impact of steatosis on midazolam elimination^38^. In contrast, Woolsey and coworkers reported that nonalcoholic steatohepatitis induced in humans caused an increase in the AUC of midazolam and delayed elimination rate^42^. However, they did not report any information regarding the zonation and distribution pattern of steatosis. These results suggest that not only the species and pattern might play a role, but also the etiology of steatosis.

In our study, periportal steatosis did not show an impact on the elimination rate of intraperitoneally injected codeine indicative of CYP2D6. In a rat model fed a 1% orotic acid-containing diet, severe steatosis induced a significant impact on AUC and elimination rate of orally administered metoprolol (drug substrate of CYP2D6), However, this effect was not evident when metoprolol was injected intravenously^50^. This observation suggests that also the route of administration may make a difference.In our statistical analysis, we observed a moderate correlation on one hand between the severity respectively extent of macrovesicular steatosis and the AUC of midazolam and codeine. In contrast, we did not observe a correlation between the severity respectively pattern of steatosis and the AUC of caffeine. Furthermore, microvesicular steatosis did not show a correlation with the AUC of any of the three test drugs. These results support that severity and pattern are relevant factors influencing selected parameters of the drug metabolism system.

Our results suggest a much higher complexity regarding the interrelationship of steatosis and drug metabolism. To the best of our knowledge, this study is the first report observing that drug metabolism is not only influenced by the presence of steatosis but also related to the severity and pattern of fat accumulation. Although steatosis did not affect the distribution pattern, the severity of periportal macrovesicular steatosis was correlated to the activity of pericentrally expressed CYP enzymes and corresponding drug elimination represented by the AUC.

In consequence, regulation of the activity of pericentrally located CYP enzymes might be influenced by factors beyond the presence or absence of fat in the pericentral hepatocytes suggesting some signaling mechanism as postulated by Ghallab in case of periportal fibrosis^8^.

As mentioned before, this finding resembles the recent finding of Ghallab and co-workers mentioned in the introduction^8^, that periportal fibrosis affected metabolic zonation similar to pericentral fibrosis. They proposed in another publication^51^ an inflammation-associated suppression of metabolic gene networks in acute and chronic liver disease. Furthermore, they observed that different types of acute and chronic inflammatory stimuli activated the same gene regulatory networks. Upregulation of inflammatory genes occurred simultaneously with the downregulation of metabolic genes. This is what Ramadori and Christ called “Molecular economy of the hepatic acute phase reaction”^52^. Assuming that even hepatic steatosis without pronounced steatohepatitis would represent a mild inflammatory stimulus might explain our observation of periportal steatosis affecting the activity of certain CYP enzymes. However, the fact that drug metabolism may be altered even in absence of morphological alterations as observed in the mouse model of chronic intermittent hypoxia, might speak for other signaling mechanisms^41^.

### Perspectives

These observations call for further and more comprehensive investigations of drug metabolism and eventual intralobular signaling mechanisms. In future studies, the assessment of drug metabolism should include all aspects including CYP enzyme distribution, expression level, activity, and pharmacokinetics. In terms of steatosis, not only etiology, severity, type, and zonal distribution of fat accumulation should be taken into account but also inflammatory mediators or other signaling molecules. This might help to better understand the currently observed controversial results.

## Methods

### Experimental design

Male C57BL6/J mice were fed for either two weeks or four weeks with a high-fat diet, low in methionine and choline content (HF-diet) In contrast to the methionine-choline deficient diet (MCD), this dietary protocol induces periportal steatosis in rats and mice, but does not cause weight loss^23,24,26,35^. The control group received a standard maintenance diet, resulting in three experimental groups (n = 4–6/group).

Hepatic steatosis was characterized histologically in respect to the pattern (micro- versus macrovesicular), zonated distribution (periportal versus pericentral), and severity. Steatosis severity was assessed based on a triglyceride assay, and automated computer-based quantitative assessment using whole slide scans.

The spatial distribution of four drug-metabolizing enzymes (CYP1A2, CYP2D6, CYP2E1, and CYP3A4) was visualized by immunohistochemistry. CYP activity was determined using five model reactions in a photometric or fluorometric assay. Pharmacokinetics was assessed using a drug cocktail consisting of caffeine, midazolam, and codeine. Injection of the drug cocktail was followed by micro-blood sampling at ten scheduled time points over six hours and subsequent ultra-performance liquid chromatography-tandem mass spectrometry (UPLC-MS/MS) analysisAnimal experiment.

### Animals

All in-vivo studies were carried out using male C57BL6/J mice (Janvier, France) 28–30 g of body weight, between eight to ten months of age (ex-breeder) (n = 4–6/group). Mice were randomly housed in groups of three animals and had free access to food and water. The cages were kept under constant environmental conditions with a twelve-hour light/dark cycle in a conventional animal facility, constant room temperature (21 ± 2 °C) and 45–65% relative humidity.

### Feeding

Mice were subjected to either 2 weeks or 4 weeks of feeding the same HF-diet (E15652-94 EF R/M, high fat MCD mod, Ssniff Spezialdiäten GmbH, Sulzfeld, Germany) to induce steatosis of different severity (for details in the composition see Supplementary Table S5). The control group of mice was supplied with the standard maintenance diet (Altromin Spezialfutter GmbH, Germany) Body weight gain and food intake were monitored daily.

### Drug cocktail injection

The drug cocktail consisted of caffeine, midazolam, and codeine (2 mg/kg body weight (bwt), 5 mg/kg bwt, and 2 mg/kg bwt, respectively) and is widely used for pharmacokinetic studies^19^, see Table 1. Each drug was diluted in 200 µl of sterile water resulting in a concentration of 1 mg/ml for midazolam and codeine, and 2.5 mg/ml for caffeine. The three aliquots were mixed resulting in a total volume of 600 µl. The volume to be applied (180 µl/30 g bwt) was calculated based on the body weight of the individual mouse.

**Table 1:** Drug concentration, dose, and source. (^a^dose per body weight; #needed volume of stock solution (µl)/100 g mouse diluted in a final volume of 200 µl; ^b^final volume of diluted drug cocktail per body weight).

CYP test drug	Company	Respective CYP isoforms	Dose^a^^19^	Concentration stock solution	Volume stock solution#	Final volume^b^
Midazolam	B. Braun	CYP3A4	2 mg/kg	5 mg/ml	40 µl/100 g	600 µl/100 g
Caffeine citrate	Cooper	CYP1A2	5 mg/kg	25 mg/ml	20 µl/100 g	
Codeine phosphate hemihydrate	Lipomed	CYP2D6	2 mg/kg	5 mg/ml	40 µl/100 g	

### Route of drug application

The drug cocktail was administered via intraperitoneal injection. We preferred this method compared to oral or intravenous injection for three reasons: first to reduce the stress for the animal caused by gavage; second, to reduce the risk of complications following intravenous injection into the penis vein; third, to reduce the risk of drug contamination upon injection and sampling from the same site, the tail vein thereby possibly causing false-positive results^53^.

### Sacrifice and sampling

Animals were subjected to repeated micro-blood sampling at ten scheduled time points over six hours (before injection, 15 min, 30 min, 1 h (h), 1.5 h, 2 h, 2.5 h, 3 h, 4 h, 6 h). Samples were collected from the tail vein using heparinized calibrated end-to-end capillaries tubes (Minicaps, Hirschmann) with a volume of 9 μl (accuracy 0.5%, CV < 1%). Animals were sacrificed 24 h after injecting the drug cocktail using an overdose of isoflurane with exsanguination.

Liver tissue samples were collected from four different liver lobes (left lateral lobe, right median lobe, right superior lobe, and inferior caudate lobe), and were subjected either to formalin fixation and paraffin embedding or were snap-frozen and cryopreserved at -80 °C until used.

### Ethics statement

Animal experiments were performed according to the current German regulations and guidelines for animal welfare and the ARRIVE Guidelines for Reporting Animal Research. The animal experiment protocol was approved by the Thüringer Landesamt für Verbraucherschutz, Thuringia, Germany (Approval-Number: UKJ-19-020).

### Histology

#### Hematoxylin–Eosin (HE) staining

Formalin-fixed samples were paraffin-embedded. Blocks were used to prepare 3 µm sections. Sections were subjected to HE-staining for assessment of steatosis. Other sections were used for immunohistochemistry to determine the spatial distribution of four different CYP enzymes. Stained sections were digitized using a whole slide scanner (L11600, Hamamatsu, Japan) equipped with the NDP.view2Plus Image viewing software (Version U12388-02) at 40 × magnification.

#### Qualitative steatosis assessment

Steatosis was assessed qualitatively in terms of type and distribution in whole slide images from HE-sections. We differentiated between micro- and macrovesicular steatosis and the mixed pattern based on the size of the lipid droplets in the hepatocytes. Furthermore, we discriminated between zonation patterns such as periportal, midzonal, and pericentral steatosis. We also looked at the intrahepatic distribution of steatosis by analyzing one section from each of the four liver lobes.

#### Image analysis-based steatosis quantification

To determine the severity of steatosis, we looked at sections from the four different liver lobes of the same animal to increase the relative sample size. We calculated the mean severity of steatosis based on the total surface covered by the individual lobes (relative surface covered by lipid droplets respectively by macro- and microvesicular steatosis).

The extent respectively the severity of steatosis were quantified using Histokat, a proprietary software based on a machine-learning algorithm created by Fraunhofer MEVIS. The algorithm divides the whole slide scan into small square tiles of a given size. Using a minimum of 30 tiles per image from the four different liver lobes on one section and different representative images of the series, the software was trained to recognize single events like the lipid droplets (event recognition algorithm) or certain patterns (pattern recognition algorithm).

**Figure 9:**
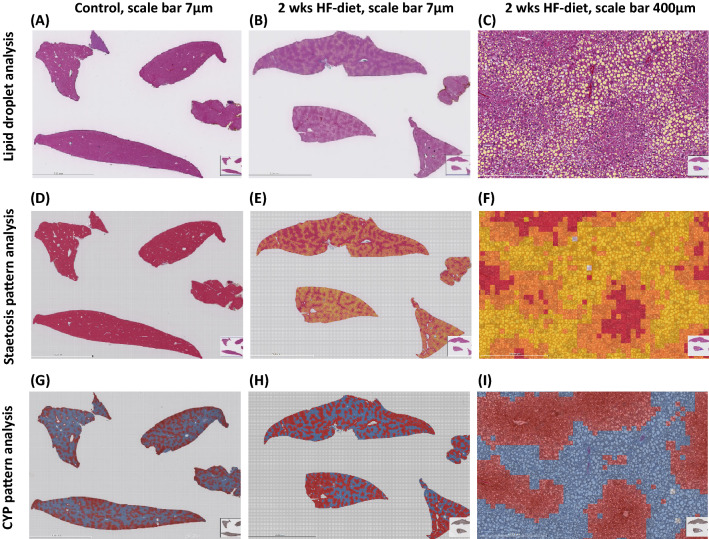
Image analysis of whole slide scan with sections from four different liver lobes using Histokat event recognition and pattern recognition algorithm; (**A**) Overlay image of normal liver after color coding of lipid droplets with yellow; (**B**,**C**) Overlay image of steatotic liver after color coding of lipid droplets with yellow; (**D**) Overlay image of normal liver after color coding the different patterns to be quantified; (**E**,**F**) Overlay image of steatotic liver after color coding the different patterns to be quantified. Square tiles classified into three color classes (orange for microvesicular steatosis, yellow for macrovesicular steatosis and red for non-steatotic hepatocytes, lumen of the vessels excluded); (**G**) Overlay images of normal liver after color coding the immunohistochemical visualization of CYP3A4 expression; (**H**,**I**) Overlay images of steatotic liver after color coding the immunohistochemical visualization of CYP3A4 expression. Square tiles classified into two color classes (blue for the CYP negative or mildly stained hepatocytesand red for strong to moderate CYP stained hepatocytes).

First, we assessed the number and relative surface in the image covered by lipids droplets, irrespective of their size, see Fig. 9B,C. For a normal liver, see Fig. 9A. Second, we used the pattern recognition algorithm (generic classification 128) to quantify the relative surface covered by fat-laden hepatocytes. Second, we determined the relative surface covered by microvesicular steatotic hepatocytes and the area covered by macrovesicular steatotic hepatocytes. Adding both together resulted in the total surface covered by steatotic hepatocytes, see Fig. 9E,F. For a normal liver, see Fig. 9D.

#### Immunohistochemistry

Immunohistochemistry was performed in 3 µm thick formalin-fixed paraffin-embedded liver tissue sections. Different CYP antibodies were used for the detection of CYP3A4, CYP1A2, CYP2D6, CYP2E1, and GS see Table 2. Liver tissue sections were deparaffinized and rehydrated using descending grades of ethanol. Then, antigen retrieval was performed with Trisodium-citrate buffer (Ph6.1) using a steamer for 30 min at 100 °C, followed by cooling for 20 min. Peroxidase blocking was applied to the tissue section for blocking the endogenous peroxidase. Ready-to-use protein block (ab64226, Abcam, Germany) was used for blocking the endogenous IgG. Tissue sections were incubated with the respective CYP antibody overnight at 4 °C, see Table 2. In the case of rabbit polyclonal primary antibodies (CYP3A4, CYP2D6 and CYP2E1), signals were amplified by applying the Rabbit-specific HRP/DAB IHC detection system (ab236469, Abcam) for 40 min at room temperature. In the case of mouse monoclonal primary antibodies (CYP1A2 and GS), the antibodies were first biotinylated using the Dako Animal Research Kit Peroxidase for Mouse primary antibody, (K3954, Dako, Denmark). In this case, additional blocking was performed using the Avidin/Biotin Blocking kit (ab64212, Abcam) before applying the biotinylated first antibody. Thereafter, the Avidin-HRP complex was added.

**Table 2:** CYP antibodies used for IHC-detection of CYP distribution and expression pattern in liver tissue sections.

Antibody	Company	Order-Nr	Dilution	Detection systems
Anti-CYP3A4 antibody-polyclonal in rabbit	Abcam, Germany	ab3572	1/2000	Rabbit-specific HRP/DAB Detection IHC Detection Kit—Micro-polymer (ab236469, Abcam, Germany)
Anti-CYP2D6 antibody-polyclonal in rabbit	Abcam, Germany	ab230690	1/3000	
Anti-CYP2E1 antibody-polyclonal in rabbit	Sigma-Aldrich, Germany	HPA009128	1/400	
Anti-CYP1A2 antibody-monoclonal in mouse	Abcam, Germany	ab22717	1/500	Dako Animal Research Kit Peroxidase for Mouse primary antibody, (K3954, Dako, Denmark)
Monoclonal Mouse Anti-GS	Merck	MAB302	1/1000	

Visualization of the reaction was accomplished by applying the DAB-chromogen for (3–5) minutes also at room temperature. Counterstaining was performed using Dako hematoxylin (CS700, Dako, Denmark) for 6 min. A negative reagent-control slide was added in each run using the same procedures without applying the primary antibody.

#### Qualitative assessment of CYP-expression

For qualitative assessment, we determined the zonal distribution and discriminated between periportal, midzonal, and pericentral localization of the CYP signal. Signal intensity was classified as mild, moderate, or strong.

#### Image analysis-based quantification of CYP-expression

For quantitation of the CYP signal, we used the same generic 128 algorithms as for steatosis to determine the relative surface covered by a given CYP signal, see Fig. 9H,I. For a normal liver, see Fig. 9G.

### Spectrophotometric assays

#### Hepatic triglyceride (TG) concentrations

Hepatic TG concentration was measured by a colorimetric method using TG quantitative assay kit according to the manufacturer's instructions (ab65336 Abcam, Germany). Lipids were extracted from 100 mg of snap-frozen liver tissue by homogenizing liver tissue samples in 1 ml 5% Igepal/double-distilled water solution with a mortar. The samples were slowly heated in a thermomixer at 95 °C for 4 min. Then, the samples were cooled-down and re-heated to solubilize all triglycerides in the solution. After centrifugation to remove any insoluble material, the supernatants were diluted 1:10 with double-distilled water. All reactions were performed in duplicates. 50 µl of the respective samples, standard and 50 µl of sample for background control were added to a transparent 96-well plate. Then 2 µl lipase and assay buffer were added for standard and sample wells, while 2 µl TG assay buffer was added to the sample background control. Then the reactions were incubated for 20 min at room temperature with constant agitation. Thereafter, the triglyceride reaction mix was added to all reaction wells, followed by incubation for 60 min in dark at room temperature with constant agitation. Output was measured on a microplate reader at OD570.

#### CYP activity

For the determination of CYP activity, the following model reactions were performed; Ethylmorphine-N-Demethylation (EMND) indicative of CYP3A activity^54^, Ethoxycoumarin-O-Deethylation (ECOD) indicative of CYP1A, 2A, 2B and 2C activity^55^, Ethoxyresorufin-O-Deethylation (EROD) indicative of CYP1A activity^56^, p-Nitrophenol-Hydroxylation (PNPH) indicative of CYP2E1 activity^57^, and Pentoxyresorufin-O-Depentylation (PROD) indicative of CYP2B activity^56^. Samples were homogenized with 0.1 M sodium phosphate buffer (pH 7.4) (1:2 w/v). The homogenized liver samples were subsequently centrifuged at 9000×*g* for 20 min at 4 °C. The 9000 g supernatants were used to assess the activities of CYPs. Protein content was evaluated using a modified Biuret method. The CYPs activity was referred to as the protein content of the 9000 g supernatant. For all model reactions, the reaction mixture contained the 9000 g supernatant, the substrate, NADPH, MgCl2, glucose-6-phosphate, and buffer. The reaction was started with the addition of NADPH and the samples were incubated at 37 °C for 5 min (EROD), 10 min (ECOD, PROD, EMND), or 30 min (PNPH), respectively. Thereafter, the reaction was stopped with the addition of either ice-cold trichloroacetic acid (ECOD, PNPH, and EMND) or methanol (EROD, PROD). Samples were then centrifuged, and the concentrations of the main metabolites were measured in the supernatant. PNPH and EMND were quantified photometrically (Spekol 1100, Carl Zeiss, Jena) by measuring the main metabolites 4-nitrocatechol or formaldehyde, respectively. The ECOD-reaction was assessed fluorometrically by quantifying the concentration of the main metabolite 7-hydroxycoumarin. For EROD and PROD the concentration of the main metabolite resorufin was also determined fluorimetrically (RF-1502, Shimadzu, Kyoto, Japan), see Table 3.

**Table 3:** Model reaction to assess CYP-activity.

Model reaction	CYP enzymes	CYP-isoforms	Measured metabolite
Ethylmorphine-*N*-demethylation (EMND)	CYP3A^54^	3A1, 3A11, 3A13, 3A16, 3A2, 3A4	Formaldehyde
Ethoxycoumarin-*O*-deethylation (ECOD)	CYP1A, 2A, 2B, 2C^55^	1A2, 2A1, 2A6, 2B1, 2B6, 2C11, 2C9	7-Hydroxycoumarin
Ethoxyresorufin-*O*-deethylation (EROD)	CYP1A^56^	1A1, 1A2	Resorufin
p-Nitrophenol-hydroxylation (PNPH)	CYP2E1^57^	2E1	4-Nitrocatechol
Pentoxyresorufin-*O*-Depentylation (PROD)	CYP2B^56^	2B1, 2B6,	Resorufin

#### Ultra-performance liquid chromatography-tandem mass spectrometry (UPLC-MS/MS)

Drug levels in terms of parent drug and its metabolites concentration were measured in heparinized whole blood, see Table 4. The analysis was performed using three dedicated sensitive UPLC-MS/MS methods on an Acquity UPLC System connected to Xevo TQ-XS or TQ-S detector (Waters, Eschborn, Germany). The 9 µl capillaries were shredded in total with an OMNI Bead Ruptor 24 (Bebensee, Germany) using a Yttrium-coated ceramic sphere and then fortified with the corresponding deuterated internal standards: ad 12.5 ng/ml midazolam-d4 and OH-Midazolam-d4 or 250 ng/ml caffeine-d9 or codeine-d6, norcodeine-d3, codeine-6-glucuronide-d3, morphine-3-glucuronide-d3 and morphine-d6 (ad 5.5 ng/ml whole blood each). After protein precipitation and liquid/liquid extraction, the diluted supernatant was injected into the UPLC system. For each test drug, a specific chromatographic method was used. A Waters CSH C18 1.7 µm, 2.1 × 150 mm column kept at 50 °C was used for midazolam/OH-Midazolam. Gradient separation was performed within 6 min. A Waters BEH-Phenyl 1.7 µm, 2.1 × 100 mm kept at 40 °C was used for caffeine gradient separation within 2.5 min. A Waters HSS T3 1.8 µm, 2.1 × 150 mm column was chosen for codeine and metabolites gradient separation at 50 °C. Chromatographic runtime was 9 min. The mass-spectrometer was operated in ESI + mode and three transitions were monitored in SRM for each analyte and two for the internal standards. The limits of quantification for caffeine was 10 ng/ml and for all other analytes at least 1.0 ng/ml. Multi-point matrix calibration was conducted for each analyte. All analytes are accredited under DIN EN ISO 15189 standard. midazolam and OH-Midazolam and the Opiates are accredited in addition for forensic purposes according to DIN EN ISO 17025.

**Table 4:** Model reaction to assess CYP-activity.

Model reaction	CYP enzymes	Measured metabolite
Midazolam	CYP3A4	OH-Midazolam
Caffeine	CYP1A2	No metabolites
Codeine	CYP2D6	Norcodeine, codeine-6-glucuronide, morphine-3-glucuronide, morphine

### Pharmacokinetic analysis

#### Area under the curve (AUC)

**Figure 10:**
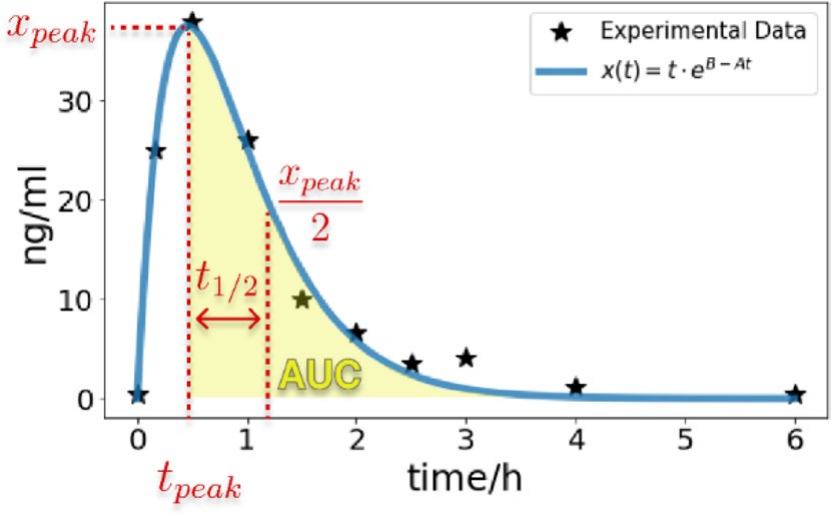
Pharmacokinetic parameters are calculated from the exponential decay curves, fitted to the experimental data. Shown are exemplary data from codeine-6-glucuronide control replicate 4 with fitted parameters A = 2.168 and B = 5.397. Extracted pharmacokinetic parameters are the peak concentration ${x}_{peak}$, time of the peak concentration ${t}_{peak}$, half-life ${t}_{1/2}$, and area under the curve from the maximum (AUC).

For each replicate, the time courses of the metabolic components after bolus injection were parameterized under the assumption that an increase to a maximum turning point is followed by an exponential decay. The function $x\left(t\right)=t\cdot {e}^{B-At}$ with parameters A and B was used to fit the experimental data. The python toolbox lmfit^58^ was used to estimate these parameters for each replicate separately via least squares minimization. From each fitted curve, the peak concentration ${x}_{peak}$, the time of peak concentration ${t}_{peak}$, the half-life ${t}_{1/2}$ and the area under the curve (AUC) was calculated, see Fig. 10.

The AUC was only calculated for the decay phase, i.e. the integral was calculated from the maximum turning point to t = 6 h of the fitted curve with scipy^59^. The peak time ${t}_{peak}$ was calculated by minimization of $-x(t)$ with the corresponding peak concentration ${x}_{peak}=x({t}_{peak})$. The half-life time ${t}_{1/2}$ was calculated as the time needed for the peak concentration to halve. For this, the equation $\frac{{x}_{peak}}{2}-t\cdot {e}^{B-At}=0$ was solved for $t$, whereby the half-life time results in ${t}_{1/2}=t-{t}_{peak}$.

#### Bayesian uncertainty quantification

The model $x\left(t\right)$ was interpreted as solution of an ordinary differential equation (ODE) $\dot{x}=(1-A\cdot t)\cdot {e}^{B-A\cdot t}$ and implemented in the Systems Biology Markup Language (SBML)^60,61^ format. A PEtab^62^ parameter estimation problem was created via yaml2sbml^63^. Maximum likelihood estimation was employed for parameter estimation. Assuming independent additive normally distributed noise $\sigma$, the likelihood function $L(\theta )$ reads.

$$L\left(\theta ,\sigma \right)={\prod }_{j=1}^{N}{\prod }_{k=1}^{T}\frac{1}{\sqrt{2\pi }\sigma }\mathrm{exp}\left(-\frac{{\left(x\left({t}_{k},\theta \right)-{m}_{j}\left({t}_{k}\right)\right)}^{2}}{2{\sigma }^{2}}\right),$$

where N is the number of replicates at T different measurement time points, $x\left({t}_{k},\theta \right)$  is the solution of the ODE and ${m}_{j}\left({t}_{k}\right)$ denotes the measurement value of the j-th replicate at the time point ${t}_{k}$. The PEtab format encodes this likelihood function and was used for a Bayesian uncertainty quantification. For the parameters A and B, a uniform prior distribution in the interval [0, 100] was used. Likewise, the prior for $\upsigma$ was uniformly chosen in the interval [0, 1000].

Parameters were sampled via Markov Chain Monte Carlo (MCMC) sampling using the pyPESTO software package^64^. The adaptive parallel tempering sampler^65^ with four chains and 200,000 samples was used to assess the posterior distribution. To investigate the convergence of the MCMC chains and to cut the burn-in samples, the Geweke test^66^ was applied. The first 100 samples were cut at least even if the Geweke test suggested a lower number to ensure that only samples from the converged chain were used for the ensemble. The convergence of the chains were checked visually and through the calculation of the Effective Sample Size (ESS). The ESS of all chains was above 13,900 samples, the detailed results are on FAIRDOMHub. A parameter ensemble was created from the parameter posterior distribution with pyPESTO and simulated via AMICI^67^. These forward simulations were used to calculate the credibility intervals of the posterior predictive distributions for the model outputs. The SBML model, the yaml file describing the parameter estimation problem, the complete sample of the parameter posterior, estimated parameters, AUC values, half-lifes and a visualization of the sampling traces can be found at FAIRDOMHub [https://doi.org/10.15490/FAIRDOMHUB.1.STUDY.1070.1].

#### Statistical analysis

A descriptive ordinary one-way ANOVA was used to detect the impact of the dietary induction protocol on TG levels, steatosis severity (relative surface covered by lipid droplets respectively by macro- and microvesicular steatosis), CYP activity and immunohistochemistry expression, and the AUC derived from the PK-analysis. Tukey’s multiple comparisons test was performed using GraphPad Prism version 9.3.1(471) for Windows, GraphPad Software, San Diego, California USA, www.graphpad.com. The data were expressed as mean ± standard deviation. Differences were considered statistically significant in the case of p-values below 0.05.

#### Correlation analysis

Pearson Correlation coefficient (r) with a 95% confidence interval and two-tailed P value was used to assess the possible linear correlation between steatosis severity in terms of lipid droplets analysis and CYP activity. Also, the linear correlation between macrovesicular respectively microvesicular steatosis and the AUC was assessed using GraphPad Prism. A linear correlation (r-value) lower than zero indicates a negative correlation, whereas an r-value higher than zero indicates a positive correlation. The correlation was considered to be strong in case of r-value ≥ 0.7, moderate in case of r-value being between 0.7 and 0.5, fair in case of r-value being between 0.5 and 0.3 and negligible in case of r-value < 0.3, irrespectively of the coefficient being positive or negative^68^.

The correlation matrix was calculated using Pearson correlation. p-Values were adjusted for multiple testing using the Benjamini and Hochberg method. Analysis was performed with R version 4.2.1 and the corrplot package.

### Institutional review board statement

The study was conducted in accordance with the current German regulations and guidelines for animal welfare and the ARRIVE Guidelines for Reporting Animal Research. The animal experiment protocol was approved by the ethics committee (Thüringer Landesamt für Verbraucherschutz, Thuringia, Germany) (Approval-Number: UKJ-19-020).

## Supplementary Information

Supplementary Information.

## Data Availability

Publicly available datasets were analyzed in this study. This data can be found here at FAIRDOMHub repository for sharing system biology research^69^ (https://doi.org/10.15490/FAIRDOMHUB.1.STUDY.1070.1).
