# Supplementary material for: Periportal steatosis in mice affects distinct parameters of pericentral drug metabolism
Source: Sci Rep. 2022 Dec 17;12:21825. doi: 10.1038/s41598-022-26483-6 (PMC9759570; doi:10.1038/s41598-022-26483-6)
Supplement: Supplementary file 1 — Supplementary Information. [file 41598_2022_26483_MOESM1_ESM.docx]

Article

**Supplementary materials**

| **Study** | **species** | **Model** | **PK** | **Activity** | **IHC** | **WB** | **mRNA** |
| --- | --- | --- | --- | --- | --- | --- | --- |
| Own study | Mouse | MCD+HF diet/4wks, NAFLD | Y | Y | Y | - | - |
| ^1^ | Human | NASH | Y | Y | - | - | Y |
| ^2^ | Human | NASH | - | Y | - | Y | Y |
| ^3^ | Human | NASH | - | Y | - | Y | Y |
| ^4^ | Human | NASH | - | - | Y | - | - |
| ^5^ | Human | Steatosis (Type: NA) | - | - | Y | - | Y |
| ^6^ | Human | NASH | - | - | - | Y | - |
| ^7^ | Human | NASH | - | - | - | - | Y |
| ^8^ | Mouse | HF diet+High fructose diet/8wks | Y | - | - | - | - |
| ^9^ | Mouse | MCD dieet/ 4wks | Y | - | - | Y | Y |
| ^1^ | Mouse | HF diet/4wks | - | Y | - | - | Y |
| ^10^ | Mouse | HF diet/ 10 wks | - | Y | - | - | - |
| ^11^ | SD rat | HF diet/ 8wks | Y | - | - | - | - |
| ^11^ | SD rat | MCD-diet/8wks | Y | - | - | - | - |
| ^12^ | SD rat | 1% orotic acid-diet (OA)/ 4-5wks | Y | - | - | Y | - |
| ^13^ | SD rat | HF diet | - | Y | - | Y | Y |
| ^14^ | Wi Rat | MCD/ 2-6wk | - | Y | - | Y | - |
| ^14^ | Wi Rat | 5% OA/ 2-6wk | - | y | - | Y | - |
| ^15^ | Wi Rat | MCD-diet/ 13wks | - | Y | Y | - | Y |
| ^16^ | Wi Rat | HF-diet/ 14wk | - | - | Y | - | - |
| ^1^ | human hepatoma cells | in vitro cellular steatosis | - | Y | - | - | Y |
| ^17^ | Human hepatocytes | in vitro cellular steatosis | - | Y | - | - | Y |
| ^18^ | Human hepatocytes | in vitro cellular steatosis | - | Y | - | - | Y |
| ^19^ | Human hepatocytes | in vitro cellular steatosis | - | Y | - | - | Y |
| ^20^ | human hepatocytes | in vitro cellular steatosis | - | - | - | - | Y |

**Table S1.** Compilation of studies investigating the impact of hepatic steatosis on drug metabolism. Those studies were mostly focusing on PK, CYP protein expression level and enzyme activity, but less on zonal distribution of CYP enzymes.

| Study | species | Model | CYP3A | CYP1A | CYP3A4 | CYP1A2 | CYP2D6 | CYP2E1 | 2A6 | 2A1 | 2B6 | 2B1 | 2C9 | 2C19 | 3A1 | 3A2 | 2C29 | 2C11 | 3A5 | 2D22 | 4A | 4A1 | 7A1 |
| --- | --- | --- | --- | --- | --- | --- | --- | --- | --- | --- | --- | --- | --- | --- | --- | --- | --- | --- | --- | --- | --- | --- | --- |
| Own  study | Mouse | MCD+HF diet | ↓ A | ↓ A | ↓ Pk  No diff H | ↓ Pk  No diff H | No diff  Pk, H | ↑A  No diff H | - | - | - | - | - | - | - | - | - | - | - | - | - |  | - |
| ^2^ | Human | NASH | - | - | ↓  A, m | ↓  W, A, m | ↓  W, m | ↓  W, m | ↑  W, A | - | ↑  W | - | ↑  W, A | ↓  A,m | - | - | - | - | - | - | - |  | - |
| ^4^ | Human | NASH | - | - | ↓  H | - | - | ↑  H | - | - | - | - | - | - | - | - | - | - | - | - | - |  | - |
| ^3^ | Human | NASH | ↓A,  No diff in m, W | - | ↓A,  No diff in m, W | - | - | - | - | - | - | - | - | - | - | - | - | - | - | - | - |  | - |
| ^1^ | Human | NASH | - | - | ↓ A, m, Pk | - | - | ↑ m | - | - | - | - | - | - | - | - | - | - | - | - | - |  | - |
| ^7^ | Human | NASH | - | - | - | - | - | ↑ m | - | - | - | - | - | - | - | - | - | - | - | - | - |  | - |
| ^6^ | Human | NASH | - | - | ↓ H | - | - | ↑ H | - | - | - | - | - | - | - | - | - | - | ↓ H | - | - |  | - |
| ^5^ | Human | Steatosis | - | - | ↓ m, H | - | - | ↑ m, H | - | - | - | - | - | - | - | - | - | - | - | - | - |  | - |
| ^1^ | Mouse | HF diet | - | - | ↓ A,m | - | - | ↑ m | - | - | - | - | - | - | - | - | - | - | - | - | - |  | - |
| ^10^ | Mouse | HF diet | - | - | - | - | - | ↑ A | - | - | - | - | - | - | - | - | - | - | - | - | - |  | - |
| ^9^ | Mouse | MCD diet | ↓  W,M | - | No diff pk | ↓  W, m,pk | No diff pk | - | - | - | - | - | No diff  pk | No diff  pk | - | - | ↓  W, m | - | - | ↓  W, m | - |  | - |
| ^13^ | SD rat | HF diet | - | - | - | ↓  W, A, m | - | No diff W,  A | - | - | - | ↓ A, m | - | - | ↓ m, A  no diff W | no diff  W, A | - | ↓  W, A, m | - | - | - | ↓  A, m | - |
| ^12^ | SD rat | 1% OA diet,  Oral metoprolol | - | - | - | - | ↓  W, Pk | - | - | - | - | - | - | - | - | - | - | - | - | - | - |  | - |
| ^12^ | SD rat | 1% OA diet,  i.v metoprolol | - | - | - | - | ↓W,  No diff. Pk | - | - | - | - | - | - | - | - | - | - | - | - | - | - |  | - |
| ^14^ | Wi Rat | MCD diet | ↓ A,  No diff. W | - | - | - | - | ↓ A, m | - | - | - | - | - | - | - | - | - | - | - | - | ↓ A,  W |  | - |
| ^14^ | Wi Rat | 5% OA diet | ↓ A,  ↑ W | - | - | - | - | ↑  W,A | - | - | - | - | - | - | - | - | - | - | - | - | No  diff |  | - |
| ^15^ | Wi Rat | MCD diet | - | - | - | - | - | ↑  A, m, H | - | ↓ A | - | - | - | - | - | ↓A | - | ↓A | - | - | - |  | - |
| ^16^ | Wi Rat | HFD diet | - | - | - | - | - | ↑H | - | - | - | - | - | - | - | - | - | - | - | - | - |  | - |
| ^17^ | Human hepatocytes | in vitro cellular steatosis | - | - | ↓  A, m | ↓  A, m | ↓  A, m | ↓  A, m | ↓  A, m | - | ↓  A, m | - | ↓  A, m | - | - | - | - | - | - | - | - |  | - |
| ^18^ | Human hepatocytes | in vitro cellular steatosis | - | - | ↓  A,m | ↓  A, m | - | ↓  A, m | - | - | - | - | ↓  A, m | - | - | - | - | - | - | - | - |  | - |
| ^19^ | Human hepatocytes | in vitro cellular steatosis | - | - | ↓  A, m | No diff | No diff | ↑  A, m | - | - | - | - | ↓  A, m | - | - | - | - | - | - | - | - | - | ↑  A, m |
| ^1^ | human hepatoma | in vitro cellular steatosis | - | - | ↓  A, m | - | - | ↑  m | - | - | - | - | - | - | - | - | - | - | - | - | - |  | - |
| ^20^ | human hepatocytes | in vitro cellular steatosis | - | ↓  m | - | - | - | ↑  m | - | - | ↑  m | - | No diff  m | - | - | - | - | - | - | - | - |  | - |

**Table S2.** Contradictory results regarding the impact of hepatic steatosis on expression and activity of selected CYP enzymes. (IHC=H, Western Blot = W, mRNA expression= m, Activity=A; pk=pharmacokinetics; blue color = ↓; light red color = ↑; yellow color= no difference)

| **Study** | **species** | **Model** | **severity** | **pattern** | **zonation** | **Drug Metabolism Assay** |
| --- | --- | --- | --- | --- | --- | --- |
| Own  Study | Mouse | MCD+HF diet | moderate | Micro and Macro | periportal | PK, IHC, Activity |
| ^4^ | Human | NASH | severe | Macro | diffuse | IHC |
| ^2^ | Human | NASH | severe | - | - | WB, Activity, mRNA expression |
| ^3^ | Human | NASH | - | Macro | - | WB, Activity, mRNA expression |
| ^1^ | Human | NASH | - | - | - | PK, Activity, mRNA expression |
| ^7^ | Human | NASH | - | - | - | mRNA expression |
| ^6^ | Human | NASH | - | - | - | IHC |
| ^5^ | Human | Steatosis | - | - | - | IHC, mRNA expression |
| ^1^ | Bl6 Mouse | HF diet | - | - | - | Activity, mRNA expression |
| ^10^ | Wild type Mouse | HF diet | - | micro- and macro | - | Activity |
| ^8^ | Bl6 Mouse | HF diet+High fructose diet | - | Micro | - | PK |
| ^9^ | Leptin deficient ob/ob Mouse | MCD/4wks | Severe | Macro | - | PK, WB, mRNA |
| ^11^ | SD rat | HF diet | mild | Micro? | Periportal? | Pk |
| ^11^ | SD rat | MCD diet | severe | Macro? | Diffuse? | PK |
| ^12^ | SD rat | 1% OA diet | severe | - | - | PK, WB |
| ^13^ | SD rat | HF diet | - | - | - | WB, Activity, mRNA expression |
| ^15^ | Wi Rat | MCD diet | severe | macro | diffuse | IHC, Activity, mRNA expression |
| ^16^ | Wi Rat | HF diet | - | macro | PC | IHC |
| ^14^ | Wi Rat | MCD diet | severe | macro | 2wks diffuse 6wks pericentral | WB, Activity |
| ^14^ | Wi Rat | 5% OA diet | severe | micro | 2wks pericentral 6wks diffuse | WB, Activity, mRNA expression |

**Table S3.** Lack of characterization of steatosis in previous drug metabolism studies (NASH= Nonalcoholic steatohepatitis; HF= High fat; MCD= Methionine choline deficient; OA= Orotic acid).

| **Group** | | **Control** | **2 Wks MCD+HFD** | **4 Wks MCD+HFD** |
| --- | --- | --- | --- | --- |
| **Midazolam** | **AUC (hr·ng/mL)** | **38.38±28.32** | **37,80±13.36** | **76,47±25.07*c** |
|  | **t1/2 (hr)** | **0.529±0.175** | **0.919±0.250** | **1.096±0.584** |
|  | **Cmax (ng/ml)** | **59.05±38.42** | **36.04±16.53** | **75.29±40.82** |
|  | **Time of Cmax (hr)** | **0.315±0.104** | **0.548±0.149** | **0.653±0.348** |
| **Caffeine** | **AUC (hr·ng/mL)** | **1999±350.4** | **1119±219.4**a** | **2434±306.6****c** |
|  | **t1/2 (hr)** | **1.501±0.307** | **0.8215±0.383*a** | **1.156±0.206** |
|  | **Cmax (ng/ml)** | **1165±227.2** | **1326±504.7** | **1833±547.4** |
|  | **Time of Cmax (hr)** | **0.894±0.83** | **0.489±0.228*a** | **0.689±0.123** |
| **Codeine** | **AUC (hr·ng/mL)** | **155.1±10.39** | **144.1±26.30** | **190.1±21.11**c** |
|  | **t1/2 (hr)** | **0.517±0.064** | **0.479±0.031** | **0.579±0.054**c** |
|  | **Cmax (ng/ml)** | **254.9±37.12** | **251.8±35.69** | **276.2±29.97** |
|  | **Time of Cmax (hr)** | **0.308±0.038** | **0.285±0.019** | **0.345±0.032** |

**Table S4.** Additional parameters of pharmacokinetic study (a = significance difference between Control vs. 2Wks HF-diet; b = significance difference between Control vs. 4Wks HF-diet; c = significance difference between 2Wks HF-diet vs. 4Wks HF-diet HF-diet)

| **Nutrients** | **Standard control diet [%]**  1320 formula, [Altromin International](https://altromin.de/produkte/standarddiaeten/ratten/1320) | **High fat diet with low methionine [%]**  **(**[**www.ssniff.de**](http://www.ssniff.de)**)** |
| --- | --- | --- |
| Dry matter | 88,7 | 96.8**%** |
| Crude protein (N x 6.25) | 19.2 | 12.9**%** |
| Crude fat | 4.1 | 15.1**%** |
| Sugar/Dextrins | Not available | 45.1**%** |
| Starch | Not available | 14.1**%** |
| N free extracts | 55% | 57.8**%** |
| Crude ash | 5.9% | 5.9**%** |
| Crude fibre | 6.1% | 5**%** |
| Methionine | 0.27% | 0.14**%** |
| Lysine | 0.81% | 1.25**%** |
| Lys : Met+Cys | Not available | 1: 0.28**%** |
| Met+Cys | Not available | 0.35**%** |

**Table S5. Composition of standard and high fat diet with reduced methionine and choline**

Interestingly, we observed striking differences in the fat distribution within the liver. We observed areas with almost no fat laden hepatocytes and areas with severe steatosis even in the same lobe and similar variations also between liver lobes, see Figure S1.

The fat-laden hepatocytes were distributed heterogeneously throughout the liver. As reported previously, we observed in mice^21^, but also rats^22^, a striking heterogeneity in the lobar distribution of hepatic steatosis on all levels, in terms of interlobular variation, lobar variation and inter-individual variation. This is not an uncommon finding and has been reported before in humans^23-25^. However, clinical reports are rather based on imaging technologies and not on histological analysis. In other words, the real extent of inhomogeneity in fat distribution in patients was not yet described in detail. According to Décarie et al. (2011), some areas of the liver are more prone to local fat accumulation, while the same areas are liable to disuse fat deposition. Steatosis follows typical patterns of distribution that might be diffuse, multifocal, subcapsular, geographic or perivascular^26^. However, the reason for the heterogeneous spatial distribution of fat in the liver is unclear. This heterogeneity obviously may pose problems when only obtaining a small biopsy from the large liver, as done clinically^27^. In this case, it remains rather unclear whether the given sample is truly representing the condition of the total organ. Therefore, we subjected tissue from four distinct liver lobes of each animal for histological analysis to reach a sound quantification. Using whole slide scans facilitated the automated analysis of these large samples.

**Figure S1.** Inhomogeneous distribution of fat-laden hepatocytes in a mouse liver (animal-ID MNT-042); (**A**) Intralobular variation: in this lobe (left lateral lobe) the relative surface covered by fat-laden hepatocytes ranged from 10%-50%; (**B**) Interlobular variation: comparing regions of interest in 3 different lobes, the relative surface covered by steatotic hepatocytes ranged from 5% in the left lateral lobe (see B2), to 20% in the right superior lobe (see B4) and 60% in the left median lobe (see B3).

| **(A)** | **(B)** |
| --- | --- |
| 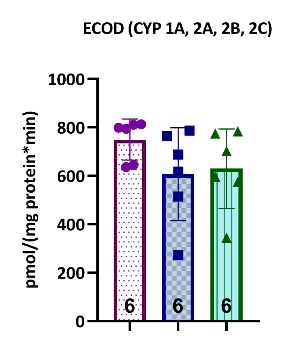 | 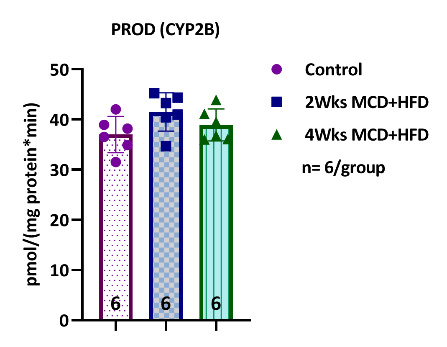 |

**Figure S2.** CYP activity. **(A)** Using the ECOD assay covering the activity of CYP1A, CYP2A, CYP2B, and CYP2C, no differences between groups were detected; **(B)** The PROD assay covering the activity of CYP2B did not show any differences between the three groups (sample size of each group displayed in the bottom of the bar).

We did not find a correlation between steatosis severity (lipid droplet, micro- or macrovesicular steatosis) and CYP2B-activity (PROD-model reaction) respectively the results of ECOD assay covering the combined activity of CYP1A, 2A, 2B, 2C see Figure S3.

|  | **ECOD (CYP1A, 2A, 2B, 2C)** | **PROD (CYP2B)** |
| --- | --- | --- |
|  | **(A1)** | **(B)** |
| **Lipid droplet** | 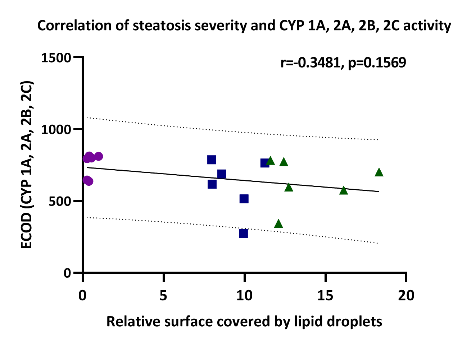 | 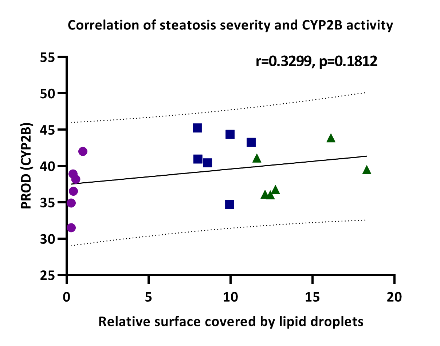 |
|  | **(C)** | **(D)** |
| **Microvesicular steatosis** | **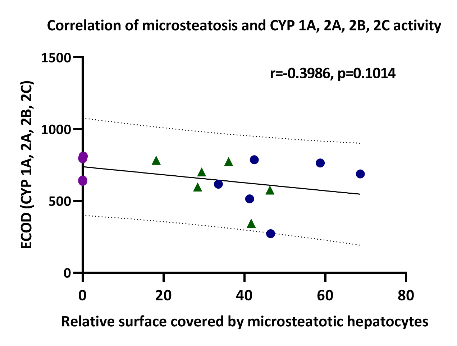** | 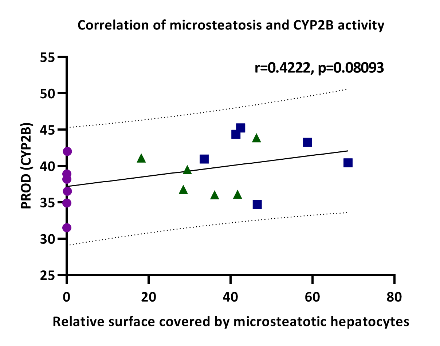 |
|  | **(E)** | **(F)** |
| **Macrovesicular steatosis** | **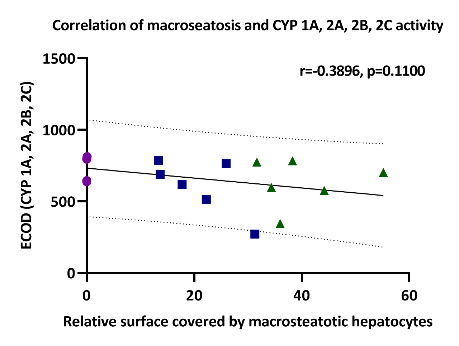** | **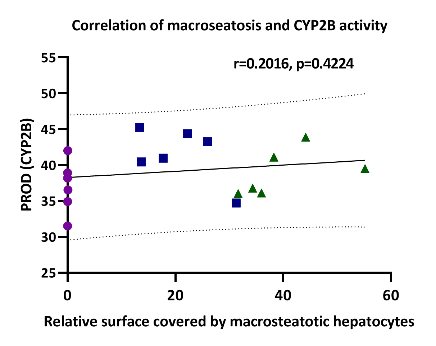** |

**Figure S3.** Correlation analysis between steatosis severity (lipid droplet analysis, micro-and macro-vesicular steatosis) and CYP activity; No correlation between (**A, B**) lipid droplet analysis, (**B, C**) microvesicular steatosis, and (**C, D**) macrovesicular steatosis and results of ECOD-model reaction determining CYP 1A, 2A, 2B,2C activity, respectively PROD-model reaction covering CYP2B. Control as magenta circles, two weeks HF-diet as blue squares, four weeks HF-diet as green triangles.

| **(A)** |  |
| --- | --- |
| 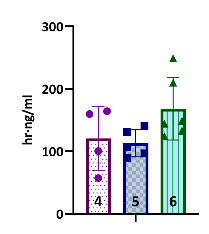**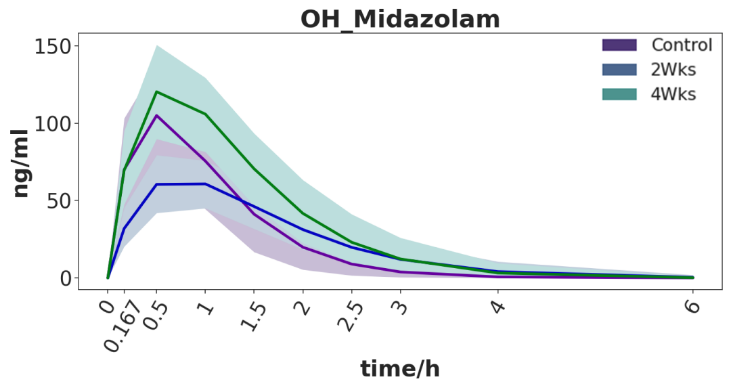** |  |
| **(B)** | **(C)** |
| 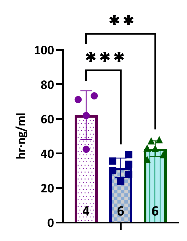**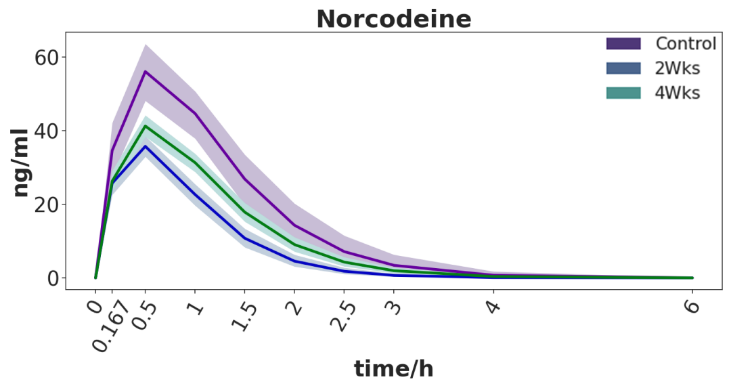** | 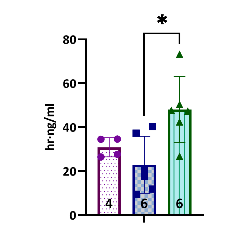**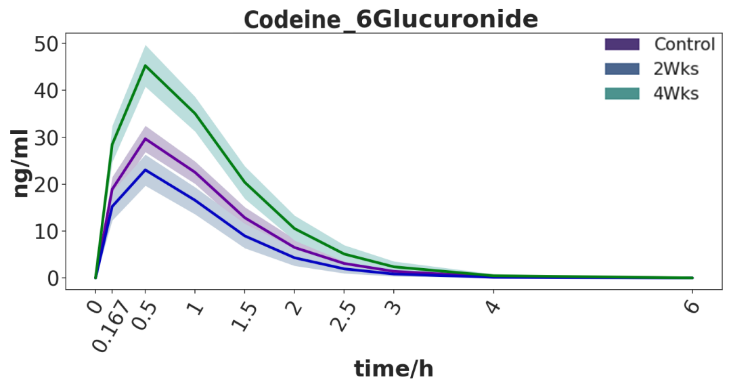** |
| **(D)** | **(E)** |
| 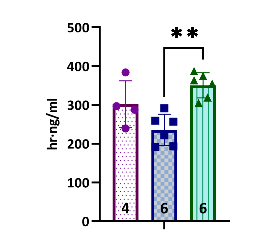**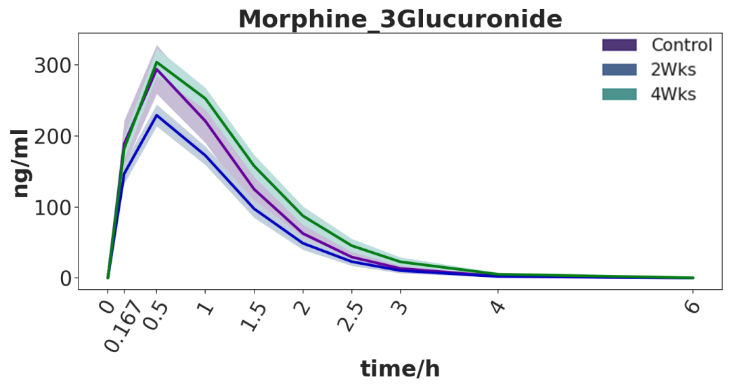** | 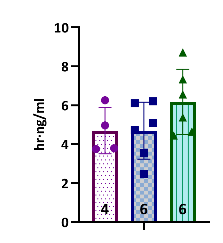**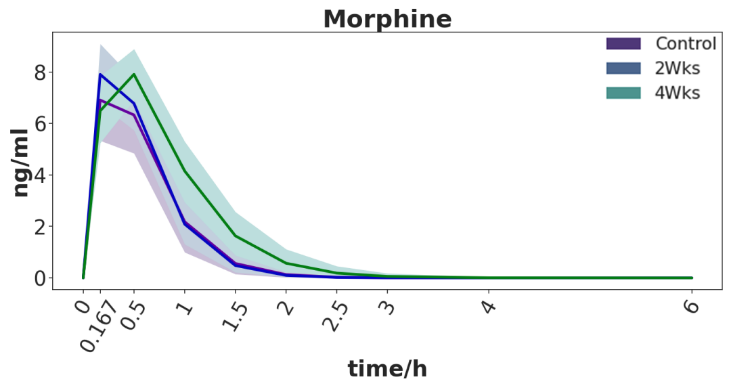** |

**Figure S4.** Drug elimination curves of the test drugs metabolites and resulting AUC. **(A)** 1-OH-Midazolam; **(B)** Norcodeine; **(C)** Codeine-6-Glucuronide; **(D)** Morphine-3-Glucuronide; **(E)** Morphine. (*significance level < 0.05, **significance level < 0.03, *** significance level < 0.0021, **** significance level < 0.0001). sample size of each group displayed in the bottom of the bar. Solid lines are mean, shaded areas correspond to the 95% credibility interval from the Bayesian analysis.

| **(A1)** | **(A2)** |
| --- | --- |
| 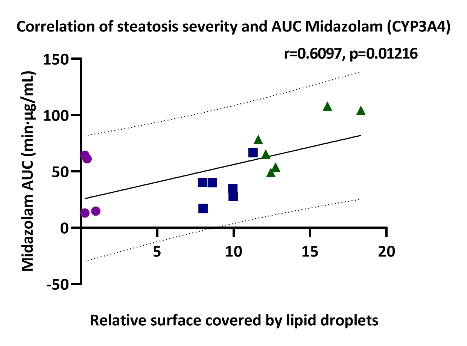 | 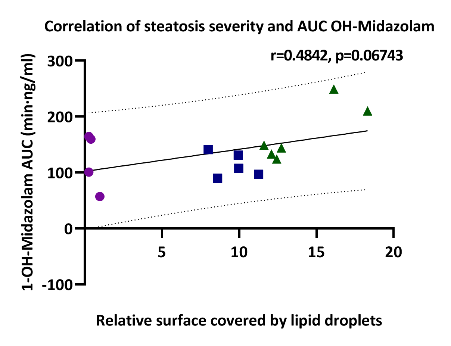 |
| **(B)** | **(C1)** |
| **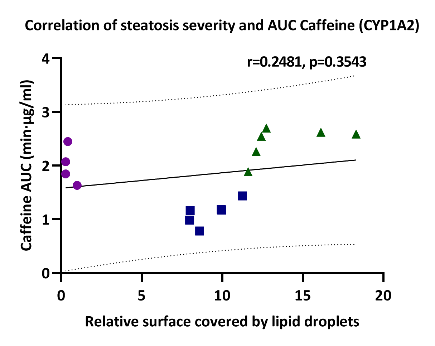** | **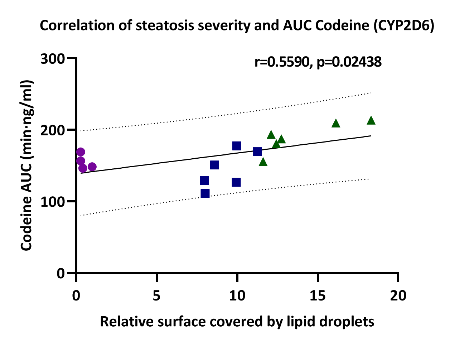** |
| **(C2)** | **(C3)** |
| 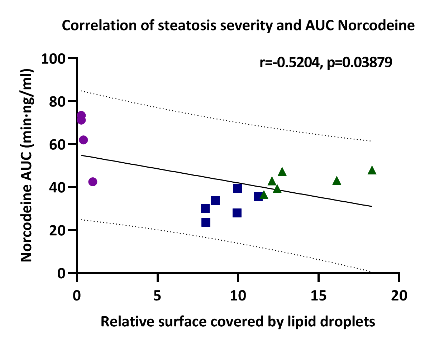 | 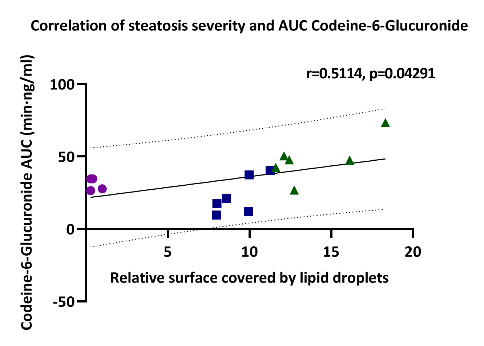 |
| **(C4)** | **(C5)** |
| **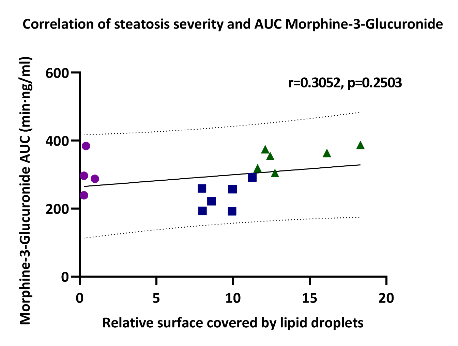** | 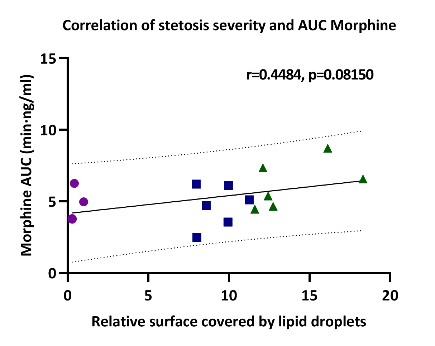 |

**Figure S5. (A1-C5)** Linear correlation between steatosis severity (lipid droplet analysis) and AUC of the test drugs and their metabolites. Moderate positive correlation between lipid droplet analysis and AUC of midazolam (CYP3A4), respectively codeine (CYP2D6), norcodeine and codeine-6-glucuronide. Correlation coefficient and p-value indicated in the figure. Control as magenta circles, two weeks HF-diet as blue squares, four weeks HF-diet as green triangles.

| **(A1)** | **(A2)** |
| --- | --- |
| 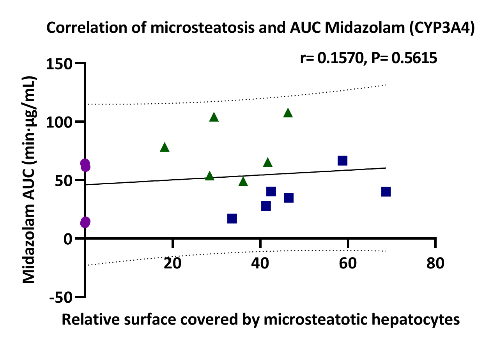 | 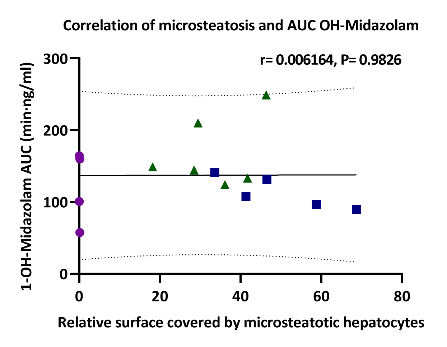 |
| **(B)** | **(C1)** |
| **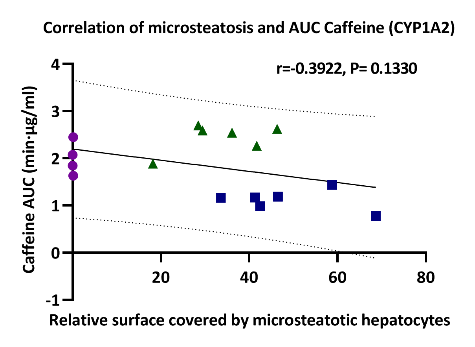** | 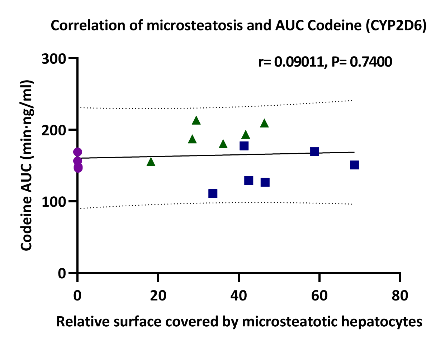 |
| **(C2)** | **(C3)** |
| 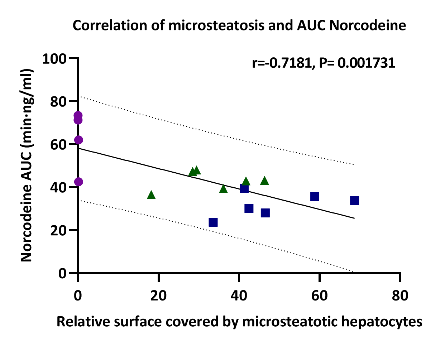 | 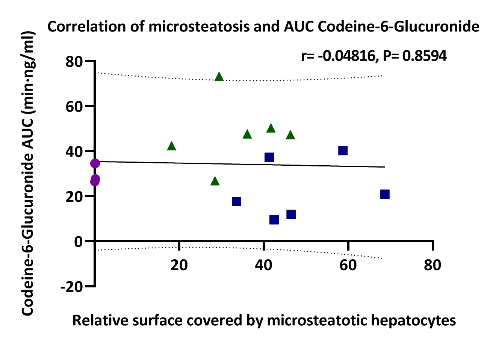 |
| **(C4)** | **(C5)** |
| **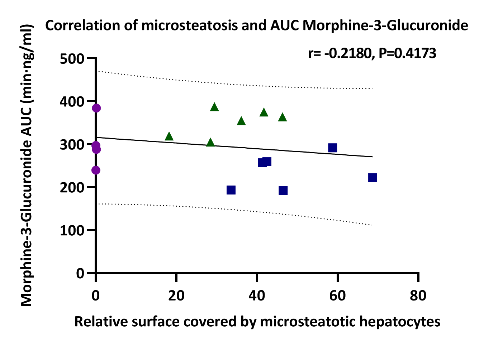** | 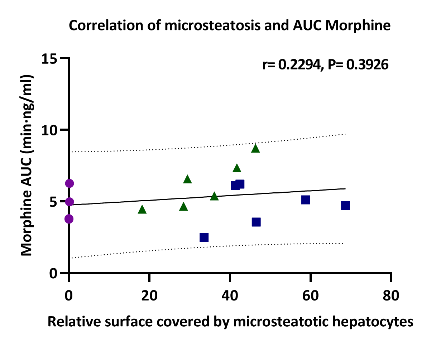 |

**Figure S6. (A1-C5)** Linear correlation between microvesicular steatosis and AUC of the test drugs and their metabolites; (**C2**) Strong negative correlation between microvesicular steatosis and AUC of norcodeine. Correlation coefficient and p-value indicated in the figure. The heavier the weight of the box line, the stronger is the correlation. Control as magenta circles, two weeks HF-diet as blue squares, four weeks HF-diet as green triangles.

| **(A1)** | **(A2)** |
| --- | --- |
| 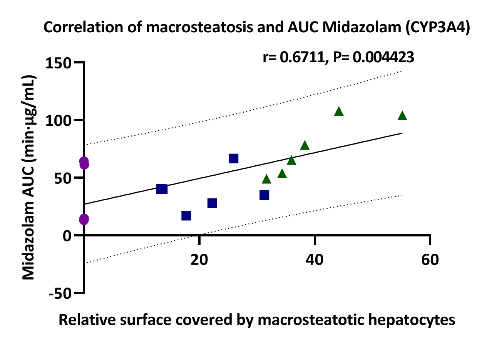 | 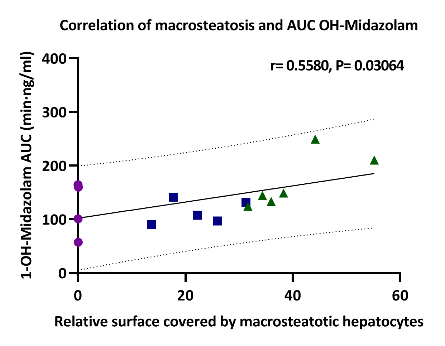 |
| **(B)** | **(C1)** |
| **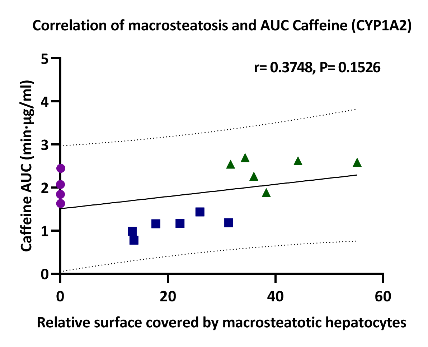** | 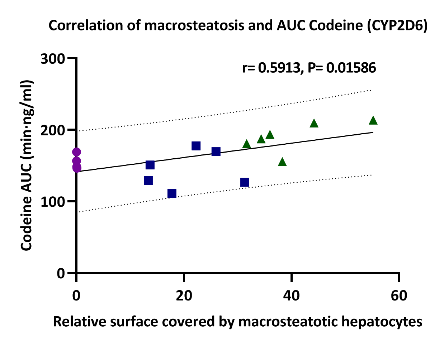 |
| **(C2)** | **(C3)** |
| **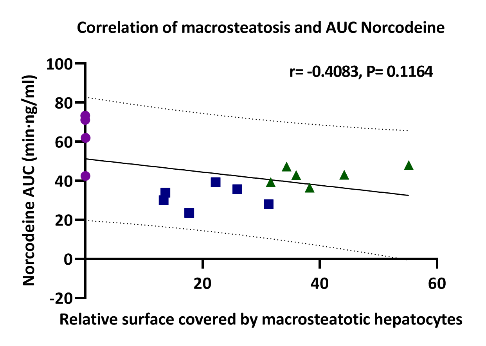** | 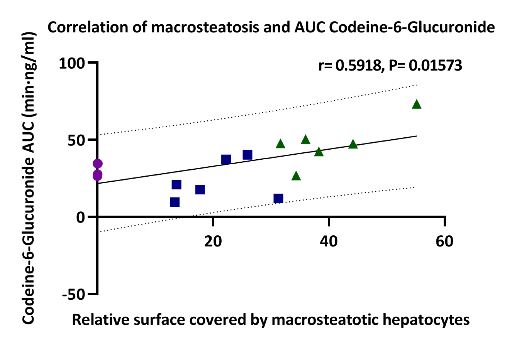 |
| **(C4)** | **(C5)** |
| **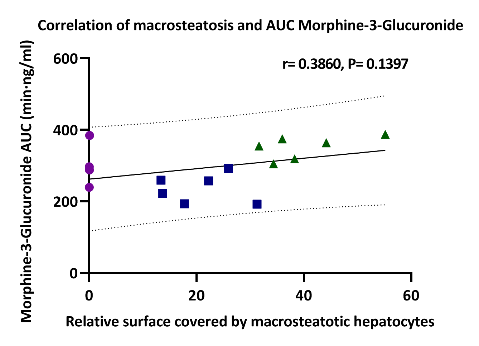** | 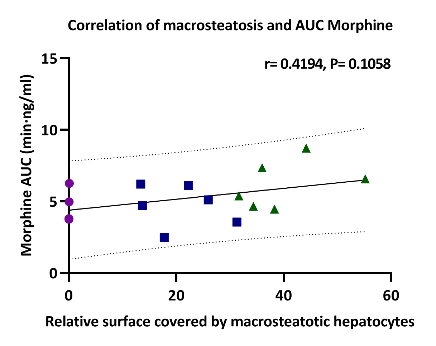 |

**Figure S7. (A1-C5)** Linear correlation between macrovesicular steatosis and AUC of the test drugs and their metabolites. Moderate positive correlation between macrovesicular steatosis and AUC of midazolam (CYP3A4) and OH-midazolam, respectively codeine (CYP2D6) and codeine-6-glucuronide. Correlation coefficient and p-value indicated in the figure. Correlation coefficient and p-value indicated in the figure. Control as magenta circles, two weeks HF-diet as blue squares, four weeks HF-diet as green triangles.

References

1 Woolsey, S. J., Mansell, S. E., Kim, R. B., Tirona, R. G. & Beaton, M. D. CYP3A Activity and Expression in Nonalcoholic Fatty Liver Disease. *Drug Metab Dispos* **43**, 1484-1490, doi:10.1124/dmd.115.065979 (2015).

2 Fisher, C. D. *et al.* Hepatic Cytochrome P450 Enzyme Alterations in Humans with Progressive Stages of Nonalcoholic Fatty Liver Disease. *Drug Metabolism and Disposition* **37**, 2087-2094, doi:10.1124/dmd.109.027466 (2009).

3 Kolwankar, D. *et al.* Association Between Nonalcoholic Hepatic Steatosis and Hepatic Cytochrome P-450 3A Activity. *Clinical Gastroenterology and Hepatology* **5**, 388-393, doi:10.1016/j.cgh.2006.12.021 (2007).

4 Weltman, M. D., Farrell, G. C., Hall, P., Ingelman‐Sundberg, M. & Liddle, C. Hepatic cytochrome P450 2E1 is increased in patients with nonalcoholic steatohepatitis. *Hepatology* **27**, 128-133 (1998).

5 Hata, S. *et al.* Cytochrome 3A and 2E1 in human liver tissue: Individual variations among normal Japanese subjects. *Life Sci* **86**, 393-401, doi:10.1016/j.lfs.2010.01.011 (2010).

6 Bell, L. N. *et al.* Bariatric surgery-induced weight loss reduces hepatic lipid peroxidation levels and affects hepatic cytochrome P-450 protein content. *Ann Surg* **251**, 1041-1048, doi:10.1097/SLA.0b013e3181dbb572 (2010).

7 Aljomah, G. *et al.* Induction of CYP2E1 in non-alcoholic fatty liver diseases. *Exp Mol Pathol* **99**, 677-681, doi:10.1016/j.yexmp.2015.11.008 (2015).

8 Kulkarni, N. M. *et al.* Altered pharmacokinetics of rosiglitazone in a mouse model of non-alcoholic fatty liver disease. *Drug Metab Pers Ther* **31**, 165-171, doi:10.1515/dmpt-2016-0008 (2016).

9 Li, H. *et al.* In vivo cytochrome P450 activity alterations in diabetic nonalcoholic steatohepatitis mice. *Journal of Biochemical and Molecular Toxicology* **31**, e21840, doi:10.1002/jbt.21840 (2017).

10 Abdelmegeed, M. A. *et al.* Critical role of cytochrome P450 2E1 (CYP2E1) in the development of high fat-induced non-alcoholic steatohepatitis. *J Hepatol* **57**, 860-866, doi:10.1016/j.jhep.2012.05.019 (2012).

11 Lickteig, A. J. *et al.* Efflux transporter expression and acetaminophen metabolite excretion are altered in rodent models of nonalcoholic fatty liver disease. *Drug Metab Dispos* **35**, 1970-1978, doi:10.1124/dmd.107.015107 (2007).

12 Bang, W. S., Hwang, Y. R., Li, Z., Lee, I. & Kang, H. E. Effects of Orotic Acid-Induced Non-Alcoholic Fatty Liver on the Pharmacokinetics of Metoprolol and its Metabolites in Rats. *J Pharm Pharm Sci* **22**, 98-111, doi:10.18433/jpps30268 (2019).

13 Zhang, L. *et al.* Diet-induced obese alters the expression and function of hepatic drug-metabolizing enzymes and transporters in rats. *Biochem Pharmacol* **164**, 368-376, doi:10.1016/j.bcp.2019.05.002 (2019).

14 Stärkel, P. *et al.* Oxidative stress, KLF6 and transforming growth factor-beta up-regulation differentiate non-alcoholic steatohepatitis progressing to fibrosis from uncomplicated steatosis in rats. *J Hepatol* **39**, 538-546, doi:10.1016/s0168-8278(03)00360-x (2003).

15 Weltman, M. D., Farrell, G. C. & Liddle, C. Increased hepatocyte CYP2E1 expression in a rat nutritional model of hepatic steatosis with inflammation. *Gastroenterology* **111**, 1645-1653, doi:10.1016/s0016-5085(96)70028-8 (1996).

16 Jiang, W., Guo, M. H. & Hai, X. Hepatoprotective and antioxidant effects of lycopene on non-alcoholic fatty liver disease in rat. *World J Gastroenterol* **22**, 10180-10188, doi:10.3748/wjg.v22.i46.10180 (2016).

17 Donato, M. T. *et al.* Potential impact of steatosis on cytochrome P450 enzymes of human hepatocytes isolated from fatty liver grafts. *Drug Metab Dispos* **34**, 1556-1562, doi:10.1124/dmd.106.009670 (2006).

18 Donato, M. T. *et al.* Effects of steatosis on drug-metabolizing capability of primary human hepatocytes. *Toxicol in Vitro* **21**, 271-276, doi:10.1016/j.tiv.2006.07.008 (2007).

19 Kostrzewski, T. *et al.* Three-dimensional perfused human in vitro model of non-alcoholic fatty liver disease. *World J Gastroenterol* **23**, 204-215, doi:10.3748/wjg.v23.i2.204 (2017).

20 Rey-Bedon, C. *et al.* CYP450 drug inducibility in NAFLD via an in vitro hepatic model: Understanding drug-drug interactions in the fatty liver. *Biomed Pharmacother* **146**, 112377, doi:10.1016/j.biopha.2021.112377 (2022).

21 Schwen, L. O. *et al.* Zonated quantification of steatosis in an entire mouse liver. *Comput Biol Med* **73**, 108-118, doi:10.1016/j.compbiomed.2016.04.004 (2016).

22 Homeyer, A. *et al.* Focused scores enable reliable discrimination of small differences in steatosis. *Diagn Pathol* **13**, 76, doi:10.1186/s13000-018-0753-5 (2018).

23 Capitan, V. *et al.* Macroscopic heterogeneity of liver fat: an MR-based study in type-2 diabetic patients. *Eur Radiol* **22**, 2161-2168, doi:10.1007/s00330-012-2468-4 (2012).

24 Choi, Y. *et al.* Heterogeneous living donor hepatic fat distribution on MRI chemical shift imaging. *Ann Surg Treat Res* **89**, 37-42, doi:10.4174/astr.2015.89.1.37 (2015).

25 Keramida, G., Hunter, J., Dizdarevic, S. & Peters, A. M. Heterogeneity of intrahepatic fat distribution determined by F-18-FDG PET and CT. *Ann Nucl Med* **30**, 200-206, doi:10.1007/s12149-015-1045-8 (2016).

26 Decarie, P. O. *et al.* Fatty liver deposition and sparing: a pictorial review. *Insights Imaging* **2**, 533-538, doi:10.1007/s13244-011-0112-5 (2011).

27 Arun, J., Jhala, N., Lazenby, A. J., Clements, R. & Abrams, G. A. Influence of liver biopsy heterogeneity and diagnosis of nonalcoholic steatohepatitis in subjects undergoing gastric bypass. *Obes Surg* **17**, 155-161, doi:10.1007/s11695-007-9041-2 (2007).
